# Bio-Based Composites with Encapsulated Phase Change Materials for Sustainable Thermal Energy Storage: A Review

**DOI:** 10.3390/polym17212925

**Published:** 2025-10-31

**Authors:** Gunasilan Manar, Mohamed Shalaby, Mohd Supian Abu Bakar, Bisma Parveez, Muhammad Imran Najeeb, Mohd Khair Hassan, Sulaiman Al-Sowayan, Mohamad A. Alawad

**Affiliations:** 1 Department of Aeronautical Engineering and Aviation, National Defence University Malaysia, Sungai Besi Camp, Kuala Lumpur 57000, Malaysia; gunasilan@upnm.edu.my; 2 Department of Electrical Engineering, College of Engineering, Imam Mohammad Ibn Saud Islamic University (IMSIU), Riyadh 11432, Saudi Arabia; myshalaby@imamu.edu.sa (M.S.); sssowayan@imamu.edu.sa (S.A.-S.); maawaad@imamu.edu.sa (M.A.A.); 3 Centre of Defence, Research and Technology (CODRAT), National Defence University Malaysia, Sungai Besi Camp, Kuala Lumpur 57000, Malaysia; 4 Department of Mechanical Engineering, Institute of Technology, Zakura Campus, University of Kashmir, Srinagar 190006, Jammu and Kashmir, India; bismaparveez.ctcz@uok.edu.in; 5 Jabatan Pendidikan Kejuruteraan, Fakulti Kejuruteraan dan Alam Bina, Universiti Kebangsaan Malaysia, Bangi 43600, Malaysia; imran.najeeb@ukm.edu.my; 6 Department of Electrical and Electronic Engineering, Universiti Putra Malaysia, Serdang 43400, Malaysia; khair@upm.edu.my

**Keywords:** thermal energy storage (TES), phase change materials (PCMs), bio-based composite materials, encapsulation, energy efficiency

## Abstract

Thermal energy storage (TES) plays a vital role in advancing energy efficiency and sustainability, with phase change materials (PCMs) receiving significant attention due to their high latent heat storage capacity. Nevertheless, conventional PCMs face critical challenges such as leakage, phase separation, and low thermal conductivity, which hinder large-scale applications. Encapsulation strategies have been developed to address these issues, and bio-based composite materials are increasingly recognised as sustainable alternatives. Materials such as lignin, nanocellulose, and biochar, as well as hybrid formulations with graphene and aerogels, show promise in improving thermal conductivity, mechanical integrity, and environmental performance. This review evaluates bio-based encapsulation approaches for PCMs, examining their effectiveness in enhancing heat transfer, durability under thermal cycling, and scalability. Applications in solar energy systems, building insulation, and electronic thermal regulation are highlighted, as are emerging AI-driven modelling tools for optimising encapsulation performance. Although bio-based PCM composites outperform conventional systems in terms of thermal stability and multifunctionality, they still face persistent challenges in terms of cost-effectiveness, scalability, and long-term reliability. Future research on smart, multifunctional PCMs and advanced bio-nanocomposites is essential for realising next-generation TES solutions that combine sustainability, efficiency, and durability.

## 1. Introduction

Thermal energy storage is key to advancing renewable energy, by addressing its intermittency, boosting energy efficiency, and reducing dependence on fossil fuels. TES supports solar energy systems, building heating, ventilation, air conditioning (HVAC), and industrial processes by storing excess energy for later use, improving grid stability and energy conservation. While sensible heat storage is the most common, due to its simplicity and low cost, latent heat and thermochemical storage offer a higher energy density and are gaining attention [1,2,3]. Furthermore, the PCMs are at the core of latent heat storage and are capable of storing and releasing thermal energy during phase transitions. Their high energy density makes them ideal for applications like concentrated solar power plants, solar thermal storage, HVAC systems, and industrial heat recovery [2,4]. This material also helps to regulate temperature in textiles, vehicles, and smart grid systems, by acting as a thermal buffer [3,4].

**Figure 1:**
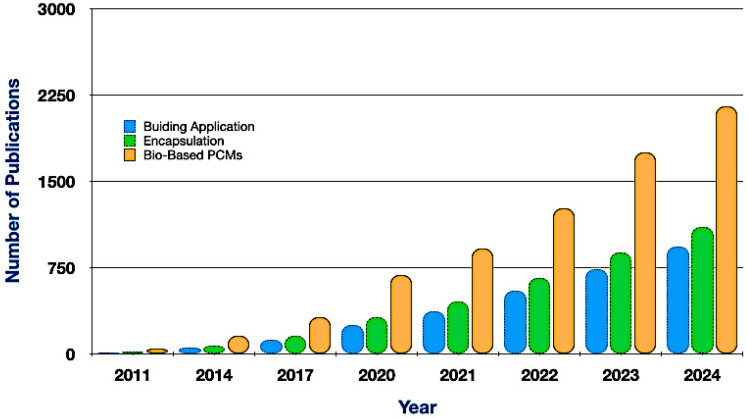
Bio-based PCMs research and development article published (source data by Google Scholar).

However, PCMs face challenges, including low thermal conductivity, phase segregation, and subcooling, which can limit performance. To overcome these issues, researchers are developing bio-based composite materials and incorporating thermally conductive additives to improve heat transfer and durability [5,6]. Cost and scalability also remain obstacles, but advancements in bio-based encapsulation and material engineering are promising [5,6,7]. Overall, PCMs play a growing role in sustainable energy systems by enabling reliable, efficient TES across diverse sectors. Thus, Figure 1 represents the summary of the growth of publications from 2011 to 2024, underscoring the heightened research interest in bio-based PCMs, encapsulation methodologies, and construction applications. Since 2020, all three domains have exhibited concurrent growth, indicating enhanced cross-disciplinary integration and an emphasis on practical implementation.

Continued innovation will be crucial for scaling TES technologies and accelerating the shift to a low-carbon energy future [1,8]. The study by Zhang et al. [9] underscored that addressing material compatibility, degradation mechanisms, and life-cycle sustainability is essential to ensure consistent performance and reduce an ecological footprint. These insights collectively underline the need for green encapsulation strategies that balance energy efficiency with environmental responsibility. Recent developments, such as vacuum impregnation, biobased matrix incorporation, and shape-stabilised composites, have been widely adopted to enhance the thermal stability, heat storage performance, and leakage resistance of PCMs. For instance, Liu et al. [7] demonstrated that the vacuum impregnation method effectively absorbs PCMs into porous natural matrices. Therefore, sustainable encapsulation materials, especially bio-based composites, improve heat transfer while reducing environmental impact [4,5,6,9].

**Figure 2:**
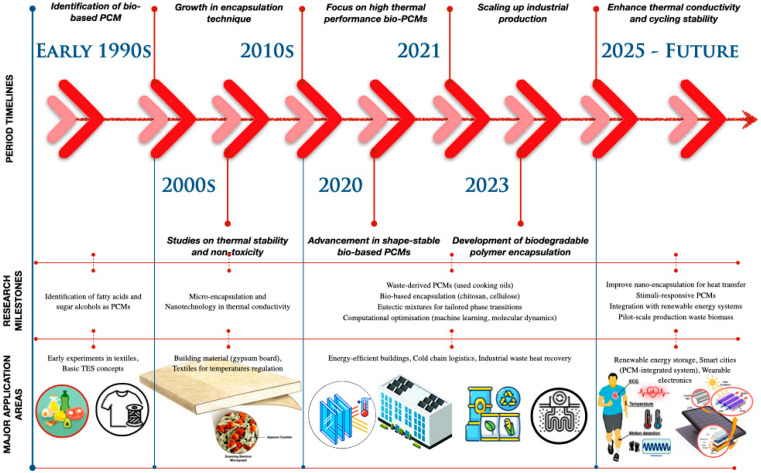
The evolution of bio-based PCMs in major application areas.

The progression of PCMs, seen in Figure 2, illustrates the evolution of bio-based PCMs, including fatty acids and sugar alcohols, which were initially identified in the 1990s, with further research in the 2000s focusing on their thermal stability and non-toxicity. The 2010s experienced advancements in microencapsulation and nanotechnology, whilst the 2020s highlighted progress in shape-stable PCMs and biodegradable polymer encapsulation. By 2023, the augmentation of industrial output broadened their applications in energy-efficient structures, cold-chain logistics, and industrial heat recovery. In anticipation of 2025, research seeks to augment conductivity, boost cycling stability, and incorporate PCMs into intelligent energy systems.

Therefore, bio-based encapsulated PCMs play a vital role in efficient, durable, and sustainable TES. Their performance depends on factors like latent heat, phase change temperature, thermal conductivity, and stability. Materials such as treated wood, natural fibres, and biodegradable polymers improve heat transfer and cycling stability. Fatty acid-based PCMs offer better oxidation resistance and thermal reliability [10,11]. Encapsulation strengthens structure and prevents leakage. Bio-based PCMs are biodegradable, have lower environmental impacts, and support CO_2_ reduction in renewable energy systems. Thus, this study aims to adopt and optimise formulations, improve encapsulation, and enhance integration with green energy and future technologies.

## 2. PCMs Properties

### 2.1. Thermal Energy Storage

**Figure 3:**
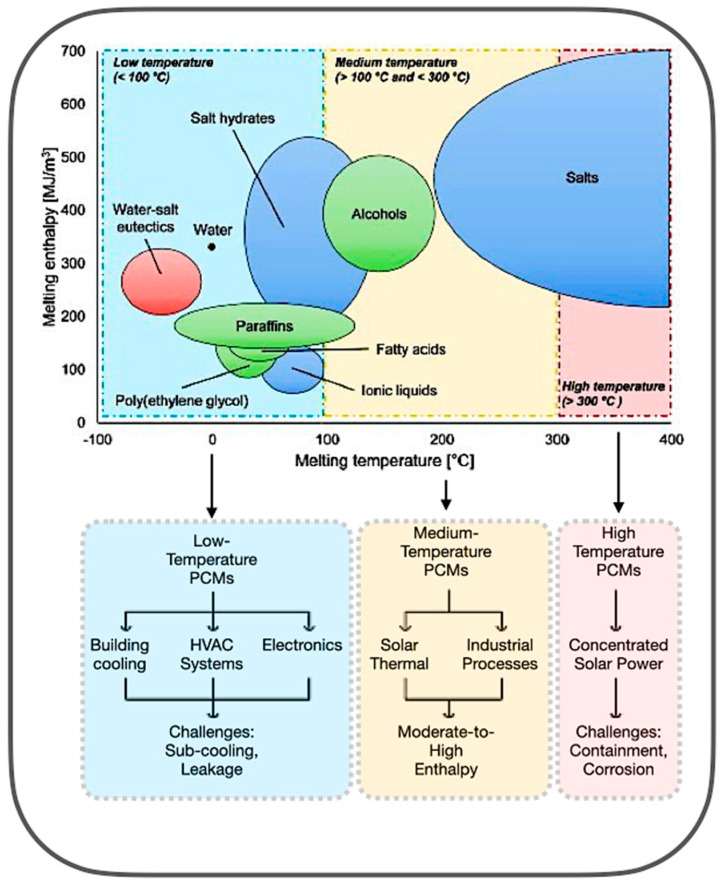
PCM melting temperature range and applications [15,16].

PCMs are key to TES, due to their ability to absorb and release latent heat during phase changes. Their effectiveness depends on the melting point, latent heat, and thermal conductivity [12,13,14]. Figure 3 illustrates that materials within the low-temperature spectrum comprise water, salt hydrates, polyethylene glycol (PEG), and paraffins. These PCMs are extensively utilised in building cooling, HVAC systems, and electronics, due to their comparatively high enthalpy values and manageable handling. Medium-temperature PCMs, such as alcohols, fatty acids, and ionic liquids, are more appropriate for solar, thermal, and industrial applications, providing moderate to high enthalpy with enhanced stability. In the high-temperature region, inorganic salts prevail, with enthalpy values rendering them suitable for concentrated solar power (CSP) applications [15,16].

### 2.2. PCMs Classification

**Figure 4:**
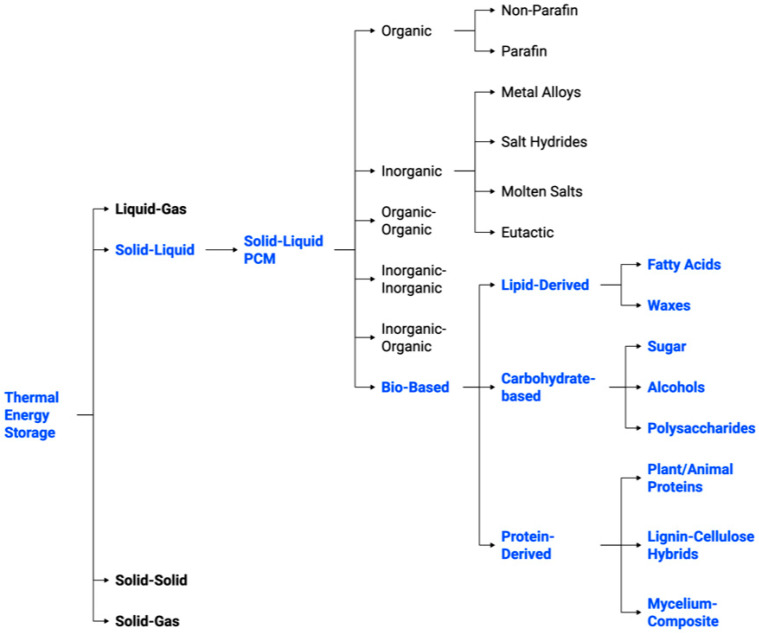
TES system classification and the PCMs subcategories [12,13,14,17].

In addition to thermal properties, PCMs can be categorised based on their material origin and composition, as depicted in Figure 4. PCMs are classified into organic, inorganic, eutectic, and bio-based categories. Organic PCMs encompass paraffins and non-paraffin organics, which are esteemed for their chemical stability, yet constrained by low thermal conductivity. Inorganics, including salt hydrates and molten salts, provide high energy density, but are prone to segregation and corrosion. Eutectics are hybrid organic–organic or inorganic–organic mixtures, facilitating adjustable melting points. Bio-based PCMs are increasingly important, due to their sustainability and diminished environmental effects.

These substances may be lipid-derived (fatty acids, waxes), carbohydrate-based (sugars, alcohols, polysaccharides), or protein-derived (plant/animal proteins, lignin-cellulose hybrids, mycelium composites). Although salts and inorganic materials provide substantial enthalpy for large-scale CSP, bio-based PCMs are more appropriate for decentralised and sustainable TES in buildings and consumer goods [12,13,14,17]. The hybrid of bio-based composites with sophisticated encapsulation methods signifies the most promising approach to achieving a balance of high energy density, cycling stability, and environmental sustainability in forthcoming TES applications.

### 2.3. Thermal Conductivity and Leakage Control in PCM Systems

**Figure 5:**
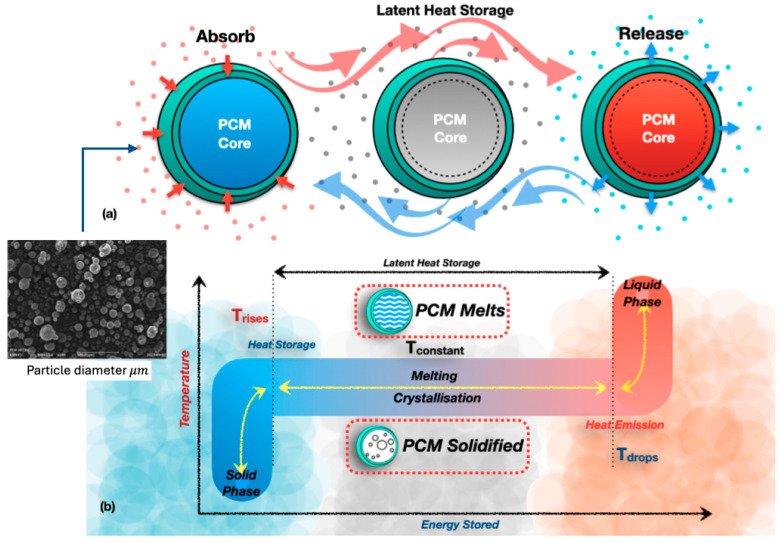
Principle behaviour of PCMs; (**a**) PCM thermal conductivity and latent heat storage, (**b**) melting and solidification behaviour of phase change material [15,18,19,20,21,22,23,24].

Thermal conductivity plays a vital role in the performance of PCMs in TES systems. It quantifies the thermal conductivity of a substance, similarly to the faster heating of a metal spoon compared to a plastic one. PCMs absorb heat upon melting and release it upon solidification. This parallels the process by which ice melts into water and subsequently refreezes. A PCM with elevated thermal conductivity facilitates rapid heat transfer, enabling quicker energy storage and release. This enhances the system’s efficiency in applications including solar heating, intelligent apparel, and energy-efficient structures. A low thermal conductivity in the PCM results in diminished heat transmission, hence impairing performance. While traditional enhancements, such as nanoparticle dispersion or hybrid TES setups, improve heat transfer, they often lead to challenges like phase separation and reduced latent heat capacity [18,19,20,21,22]. Figure 5 illustrates the fundamental behaviour of PCMs. During heating, PCMs absorb energy at a nearly constant temperature as they melt, storing latent heat. During cooling, crystallisation releases this energy, which enables stable temperature regulation compared to sensible heat storage [18,23].

Meanwhile, Table 1 highlights the thermophysical properties of organic, inorganic, eutectic, and bio-based PCMs, focusing on latent heat capacity, thermal conductivity, melting range, and stability that guides PCM selection for TES applications. Organic PCMs, such as paraffins and fatty acids, are reliable and widely used in HVAC and building applications [25,26,27,28]. Inorganic PCMs, particularly salt hydrates, deliver high latent heat, enabling rapid energy storage and release, but issues of corrosion and phase separation reduce long-term stability. Eutectic PCMs balance the properties of organics and inorganics, offering predictable melting points (20–80 °C) and moderate storage capacity, though immiscibility and leakage remain challenges [29,30,31]. Bio-based PCMs, derived from renewable resources, provide moderate heat storage with eco-friendly benefits, but they share the low conductivity limitations of organics [32,33,34,35,36].

**Table 1:** Thermophysical properties of various PCM types for TES.

PCM Type	Thermophysical Properties							References
	Latent Heat Capacity	Thermal Conductivity	Melting Point	Thermal Stability	Phase Change Leakage	Cooling	Corrosion	
Organic PCMs	Degrade over cycles Enhances TES density	Low 0.2–0.4 W/m·K Limits heat transfer Efficiency and quick energy release	18–60 °C Building/HVAC applications	Reliable over multiple cycles	Can leak if not encapsulated properly Reduces effectiveness and safety	-	-	[25,26,27,28]
Inorganic PCMs	High 100–300 J/g Suffer from phase separation High storage density and heat capacity	High 0.5–4.0 W/m·K Fast heat transfer Ideal for solar/PV cooling	-	-	-	Delays crystallisation Limits repeatable performance	Salt hydrates can corrode metal containers Affects system longevity	[29,30,31]
Eutectic PCMs	100–200 J/g Balanced performance from organic–inorganic synergy	-	20–80 °C Specific application temperatures	Important for predictable performance	Immiscibility or leakage during phase transition Limits performance over cycles	-	-	[14,37,38,39]
Bio-based PCMs	Moderate 150–220 J/g Degrade over cycles Eco-friendly TES applications Buildings and agriculture	Low 0.2–0.4 W/m·K Application in high-flux environments	20–90 °C Enhanced thermal performance	-	-	-	-	[32,33,34,35,36]

In addition, studies demonstrate that superior PCM performance emerges from the optimisation of composition and its preparation parameters. Naresh et al. [32], in their microencapsulation work, stated that the crystalline nature of the bio-based PCM chain and the integration of amorphous shell materials achieved latent heat potentials of 47.31 J/g. Also, the study by Mehrizi et al. [33] revealed that the PCM’s microstructure, made of fatty acids (e.g., palmitic acid), esters, polyethylene glycol (PEG), natural oils, and waste oils, enhances its ability to store and release heat, compared to petroleum-derived paraffins. Otherwise, the material preparation process using the accelerated thermal cycling (2000 cycles) technique by Boussaba et al. [34] has succeeded in enhancing the PCM’s thermal stability and phase change behaviour. Besides that, the study by Pundienė et al. [35] also showed that preheating the bio-composite within the temperature range of 35–60 °C before PCM integration enhanced the heat transfer characteristics and contributed to an increase in heat capacity by up to 34.8%.

## 3. Bio-Based PCM Encapsulation Composite Materials

### 3.1. Bio-Based Composite Material

Bio-based composite materials, such as natural fibres, wood-based composites, cellulose, lignin, starch, and biodegradable polymers, are gaining attention as sustainable alternatives to petroleum-based materials [40,41,42,43,44,45]. These materials offer benefits like biodegradability, strong adhesion, and environmental compatibility, making them ideal for TES applications [41,42]. Therefore, this section will discuss how natural reinforcements like cellulose, lignin, and starch-based materials enhance the mechanical and thermal stability of PCM encapsulation systems [40,42,45]. Traditional PCMs, such as paraffin-based materials, exhibit high latent heat, making them efficient for energy storage density. However, they are limited by their low thermal conductivity, which slows charging/discharging and reduces performance in high-flux applications, such as solar or electronic cooling. The performance discrepancies between the various encapsulation materials shown in Table 2 are mostly due to their intrinsic molecular structure, bonding nature, and interfacial heat-transfer behaviour. Composites’ thermal conductivity and latent heat storage capacity are determined by their structural and chemical properties. Thermal conductivity and latent heat vary between encapsulation materials, due to variances in molecular structure, bonding nature, and interfacial heat transmission.

Furthermore, Table 2 specifies that lignocellulosic materials have an improved performance due to their hierarchical fibre network, which allows for PCM infiltration and directional heat transfer [57,58,59,60,61]. Surface modification (such as silane treatment) increases endurance and PCM compatibility. Silica shells have the highest conductivity and improved thermal stability because of their dense Si-O-Si network, which improves phonon propagation [62,63,64,65,66]. However, their brittleness and incapacity to degrade limit their long-term viability.

Meanwhile, polystyrene and PMMA have higher processability and leakage resistance, but their non-polar, low-density polymer architectures result in inferior conductivity [67,68,69,70,71,72,73,74,75]. PMMA improves the encapsulation efficiency, but is UV-sensitive. Overall, bio-based encapsulants (starch, chitosan, and lignocellulose) are sustainable and have promising latent heat performance, but they must be modified to improve conductivity. Inorganic and synthetic materials (silica, polystyrene, and PMMA) offer greater stability and heat transfer, but lack biodegradability.

Hence, the hybrid systems that combine bio-based matrices with conductive nanofillers or inorganic shells provide an ideal balance for effective, long-lasting, and environmentally friendly thermal energy storage. Furthermore, future research should seek to develop a systematic relationship between material design strategies, scalable production methodologies, and application readiness assessment. Such integration will hasten the transition of bio-based PCM technologies from laboratory development to industrial use.

### 3.2. Natural Fibre-Reinforced Microcapsules

Natural fibre-reinforced encapsulated PCMs hold significant potential for sustainable TES applications, especially within the building and electric mobility industries. These systems utilise bio-based composites, such as hemp, kenaf, and flax, providing ecological benefits, superior latent heat retention, and increased thermal conductivity. Recent advancements, such as silane-treated fibres and lignin-coated structures, illustrate the capacity to address conventional PCM challenges, such as leakage, inadequate heat conductivity, and structural deterioration [52,53,54,55,56,57,58,59,60,61,76,77,78]. Translating these materials from laboratory environments to scalable, practical applications necessitates a comprehensive understanding of fibre–matrix interactions, morphology, hybridisation, and processing methodologies.

**Figure 6:**
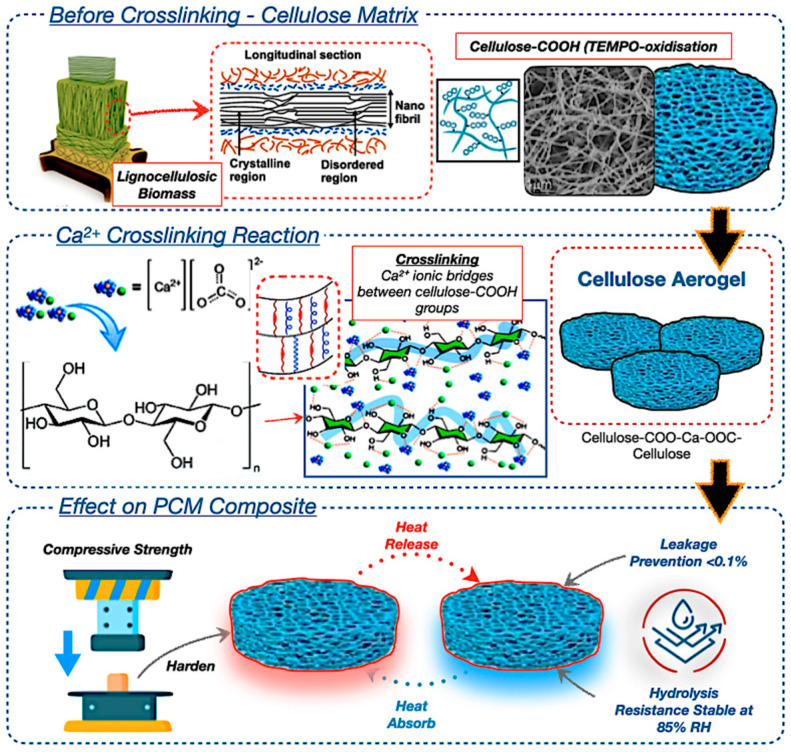
Schematic process of bio-based fibre-reinforced PCM encapsulation technique [6,36,79,80,81,82].

Figure 6 presents a schematic that depicts the principal mechanisms of hemp, kenaf, and flax fibres, which are utilised as porous scaffolds in phase change material encapsulation. The illustration emphasises fibre orientation for directional heat conductivity, surface modifications (such as silane and lignin coatings) for moisture and thermal stability, and hybridisation with nanofillers (including graphene and biochar) for improved multifunctionality. Its demonstrated applications encompass self-regulating electric vehicle batteries, biophilic building facades, and phase change material-infused insulating panels for net-zero energy structures. Scalable fabrication technologies are yet inadequately developed, with approaches like spray drying and 3D printing of fibre scaffolds demonstrating potential while experiencing limited throughput and significant energy requirements [6,36,79,80,81,82].

In addition, the interfacial connection between the fibre and matrix is crucial for the long-term stability and thermal performance of PCM. Nonetheless, inadequate dispersion or inconsistent fibre diameters may result in aggregation, diminishing performance, and challenging repeatability in scaled applications [83,84]. The incorporation of nano-fillers into fibre matrices provides multifunctional advantages, including increased conductivity, self-healing properties, and enhanced durability. Consequently, challenges existed regarding the incorporation of nano-fillers into fibre matrices, including ensuring uniform dispersion of nanofillers, preventing nanoparticle aggregation, and addressing environmental hazards during degradation [76,77,78].

### 3.3. Wood-Derived Aerogels

**Figure 7:**
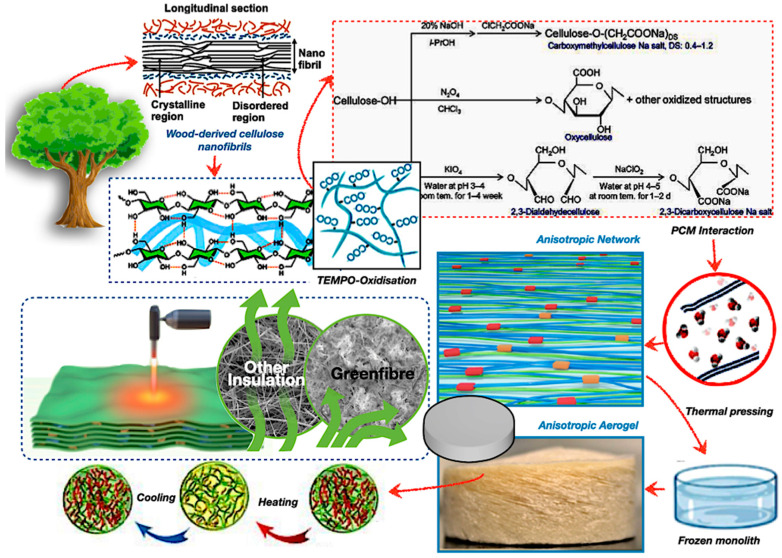
Wood-based composite material in PCM encapsulation with TEMPO-oxidised cellulose aerogel structure [93,94,95,96,97,98].

Bio-based composite materials from wood-based composites, including starch- and lignin-based adhesives, are being developed to replace synthetic resins in PCM systems [41,43,44]. Wood-derived aerogels, as potential encapsulation matrices, exhibit significant potential due to their anisotropic porosity topologies, biodegradability, and compatibility with various types of PCMs. Figure 7 illustrates thermal pressing and nanostructure alignment, which improve the endurance of the composite PCM capsules. The integration of bio-epoxy binders or reinforcement agents, such as honeycomb structures, enhances resistance to thermal fatigue, attaining encapsulation efficiency after prolonged cycling [85,86]. The utilisation of synthetic crosslinkers may compromise overall biodegradability, leading to a preference for natural alternatives, such as citric acid or tannin-based resins. Moreover, Figure 7 also highlights the possible use of aerogels in flexible insulation systems, green fibre matting, and transparent thermal windows. These materials facilitate passive thermal regulation by adaptively responding to variations in an ambient temperature [87,88,89].

Likewise, the lignin serves a dual function in wood-based aerogels, enhancing structural stiffness while hindering efficient delignification. The targeted elimination of lignin through TEMPO oxidation results in the creation of highly organised anisotropic networks of cellulose nanofibrils, which demonstrate directional thermal conductivity and facilitate substantial latent heat storage. The anisotropic aerogel architecture effectively regulates PCM leakage by refining the pore size distribution, ensuring durable long-term use. The research underscores the prospective utilisation of aerogels in adaptive architectural systems, including flexible insulation, eco-friendly fibre matting, and transparent thermal windows [90,91,92]. These materials facilitate passive thermal regulation by adaptively responding to ambient temperature variations, resulting in a reduction in energy use in net-zero energy structures [86,88,90]. However, obstacles including scalability, expense, and mechanical vulnerability persist; thus, this will enhance the broader field of future research trajectories for bio-based smart TES systems for architectural and aerospace applications.

### 3.4. Mycelium-Based Encapsulation

Mycelium matrices serve as encapsulation platforms for PCMs, representing a bio-inspired advancement in sustainable TES technologies [40,45]. The chitin–glucan structure of fungal hyphae provides inherent self-assembly, autonomous repair, and biodegradability, rendering them viable options for lightweight and adaptable energy storage devices [99,100,101]. Nonetheless, their thermal deterioration threshold constitutes a substantial limitation, restricting applications in high-temperature TES systems. The hybrid of mycelium encapsulation with UV-blocking nanoparticles, fire-resistant chitin–glucan shells, and bio-silica structures underscores a novel paradigm of multifunctional, environmentally sustainable TES devices.

**Figure 8:**
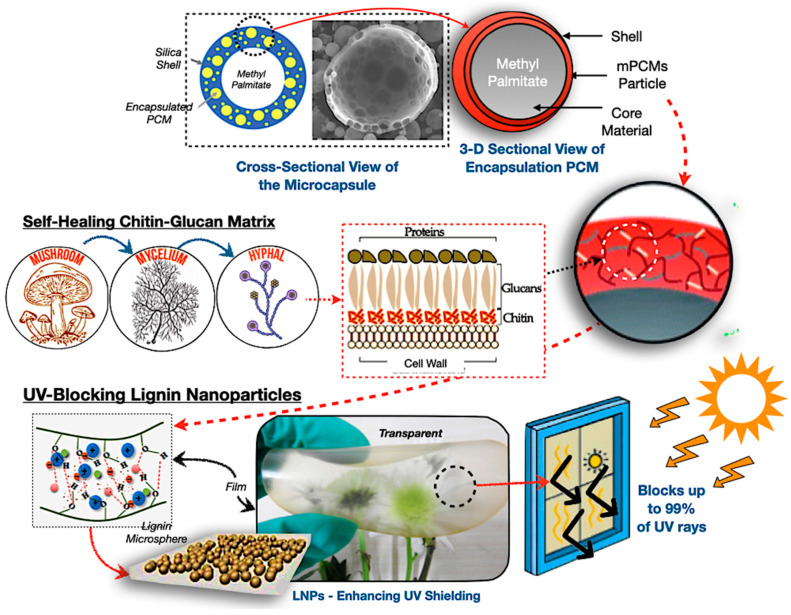
Mycelium-based PCM encapsulation mechanism [104,105,106,107,108,109].

Figure 8 depicts the mechanisms of encapsulation for mycelium-based PCMs, where the illustration represents a core–shell configuration of methyl palmitate phase change material enclosed inside silica and chitin–glucan matrices. Self-healing behaviour is facilitated by the regeneration of fungal hyphae, whereas lignin nanoparticles offer ultraviolet protection by obstructing UV radiation and improving the durability of composites in solar-exposed TES systems [102,103]. Nonetheless, substantial issues remain, including the rapid growth of genetically modified fungi, the standardisation of cultivation conditions, and the assurance of mechanical reliability during subsequent phase transitions, while these composites are validated for practical scalability and potential ecological effectiveness.

Furthermore, the structural reinforcement, including bio-silica hybridisation or epoxy densification, is essential to improve thermal durability. The thermal and structural efficacy of mycelium encapsulation is affected by the composition of the growing substrate, where nutrient-dense, lignocellulosic substrates yield denser hyphal networks, enhancing PCM encapsulation effectiveness and minimising leakage. Genetically modifying the chitin–glucan ratio may enable customised compatibility with lipid-based PCMs [99,100,101]. Mycelium-based PCM capsules have exceptional environmental performance, entirely biodegrading within 60 days in soil conditions, although they may jeopardise material durability in moist or microbially abundant settings [100,101]. To achieve a balance between biodegradability and stability, the use of protective barriers like lignin nanoparticles may be essential.

### 3.5. Bio-Based Adhesive

Cellulose and its derivatives are increasingly acknowledged as adaptable components for encapsulating PCMs in TES systems. These systems are regulated by the synergistic interplay of chemical crosslinking, rheology, and process flexibility. Citric acid and genipin exemplify divergent methodologies for crosslinking in cellulose-based systems, with genipin offering enhanced strength at a higher cost. A hybrid strategy integrating economical citric acid with superior genipin may provide both durability and cost-effectiveness, exemplifying a practical solution for industry implementation [110,111,112,113,114,115].

**Figure 9:**
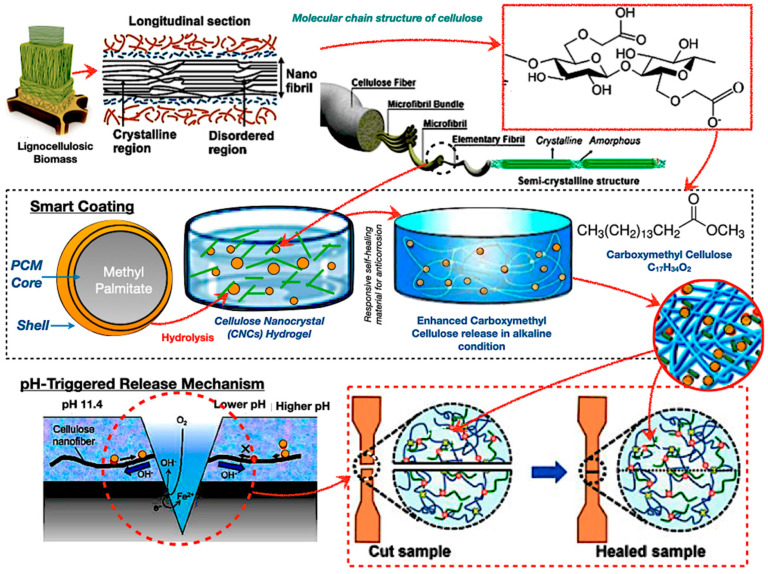
Bio−based adhesive in PCM encapsulation with pH-responsive methyl palmitate PCM core [110,111,112,113,114,115].

Figure 9 illustrates cellulose-derived smart coatings and hydrogels employed for phase change material encapsulation in TES systems. The hierarchical arrangement of cellulose facilitates its functional transformation into cellulose nanocrystals and carboxymethyl cellulose hydrogels. The primary illustration depicts a core–shell capsule, featuring methyl palmitate as the core, encased in a cellulose-derived shell that reacts to alkaline environments for regulated release. Meanwhile, the lower panels demonstrate a self-healing mechanism, offering a durable foundation for sophisticated TES applications. The self-healing capacity of cellulose-based adhesives offers exciting possibilities for extending PCM capsule lifespans. However, TES systems impose harsher conditions than biomedical or electronic contexts, and reversible bonding alone may not provide sufficient resilience under fatigue.

### 3.6. Biopolymer and Biodegradable Polymer

**Figure 10:**
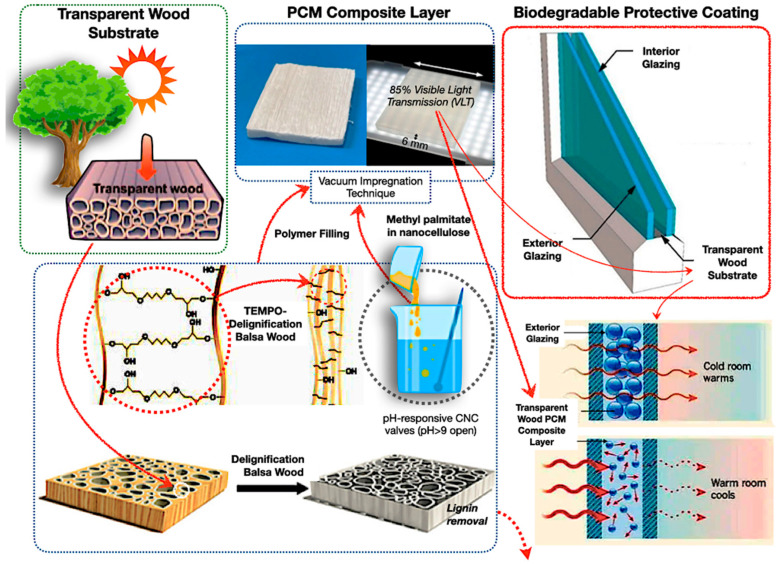
Biopolymer and biodegradable polymer in PCM encapsulation with transparent bio-PCM window design [116,117,118,119,120,121,122,123].

Bio-based encapsulated PCM composites have surfaced as a viable approach for sustainable TES. Biopolymers such as polylactic acid (PLA), polyhydroxyalkanoates (PHAs), chitosan, and cellulose-derived substrates provide biodegradability, ecological friendliness, and versatile design possibilities. Transparent wood substrates infused with PCMs, coupled with biodegradable coatings, illustrate the engineering of natural materials into high-performance TES composites for construction and industrial uses [116,117,118,119,120,121,122,123]. The efficacy of these systems is determined by intricate interactions among polymer molecular architecture, degradation characteristics, and multifunctionality in fluctuating heat conditions. Figure 10 describes transparent wood-based phase change material composites for energy-efficient glazing. Natural balsa wood undergoes delignification and TEMPO oxidation, resulting in a clear substrate. The substrate contains methyl palmitate phase change material within nanocellulose, creating a composite layer equipped with pH-responsive cellulose nanocrystal valves.

A biodegradable protective coating and glazing layers improve durability and environmental efficacy. Otherwise, the synthetic polymers often excel at durability and temperature resistance in severe settings; nevertheless, their environmental impact restricts their long-term sustainability [106,124,125]. Similarly, Okolie et al. [126] have examined the stability and safety deficiencies in traditional PCMs, emphasising bio-epoxy reinforcements for enhanced fatigue resistance, aerogels for adaptive systems, and self-healing composites for ultraviolet durability, while advocating for research to address scalability challenges for effective renewable storage. In addition, the transparent wood composites demonstrate that bio-based methods can offer sustainable thermal regulation; nonetheless, their long-term durability under actual cycling conditions necessitates additional research. The molecular weight and crystallinity of biopolymers are crucial in ascertaining heat stability and phase change material retention. Therefore, Chen et al. [127] investigate ethylene vinyl acetate (EVA) copolymer heat-storage coatings, including in situ, which attain an enthalpy, a thermal stability over 200 cycles, and flame retardancy; hence, increased molecular weights in EVA diminish chain mobility while improving crystallinity, enhancing dimensional stability for resilient protective coatings in energy-efficient structures.

## 4. Enhancement Techniques for Encapsulation PCMS

PCMs are key for TES, but their use is limited by leakage, low conductivity, and cycling instability. Encapsulation improves stability, durability, and heat transfer through three primary approaches: microencapsulation, macroencapsulation, and composite encapsulation. Otherwise, hybrid strategies, such as reinforcing microcapsules with bio-based shells or embedding macroencapsulated PCMs into conductive matrices, are promising [128,129,130,131,132]. To further enhance efficiency, advanced fillers and nanostructures are integrated into encapsulated PCMs. MXene, carbon-based nanomaterials, metallic nanoparticles, and aerogels improve thermal conductivity, enthalpy retention, and cycling stability [133,134,135,136].

**Figure 11:**
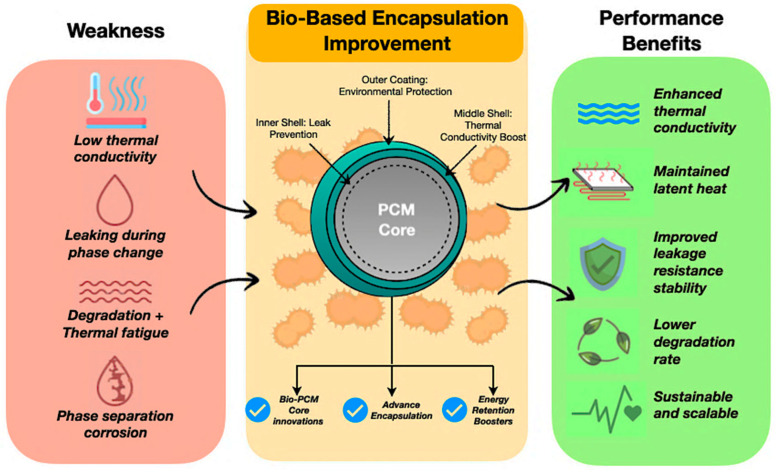
Improvement technique of bio-based PCMs [141,142].

The strategies for improving bio-based encapsulated PCMs address conventional performance limitations. Figure 11 illustrates how bio-based encapsulation techniques mitigate key deficiencies of traditional PCMs, including low thermal conductivity, leakage, thermal degradation, and phase separation. Multi-layered bio-based shells enhance stability and durability while contributing to environmental sustainability. The inclusion of hybrid nanoparticles within nanofluids further improves thermal conductivity and overall system reliability [131,133,134,135,136,137,138,139,140]. Therefore, the section below discusses the encapsulation techniques to ensure PCM containment, while enhancement techniques improve thermal efficiency and durability.

Additionally, Table 3 presents the comparative analysis of the micro-, macro-, and composite-encapsulation techniques employed to enhance the performance of PCMs in TES systems and relevant ASTM/ISO test methods for performance evaluation. Each approach has unique structural, functional, and practical advantages. Microencapsulation offers superior leakage avoidance and thermal responsiveness; yet, it is constrained by elevated costs and production intricacies. Macroencapsulation provides scalability and facilitates seamless integration into extensive systems; however, its thermal transfer efficiency is rather low. Composite encapsulation optimises performance and stability by integrating phase change materials with conductive or porous substances, enhancing thermal conductivity and mechanical resilience. The selection of technique is contingent upon the intended application: microencapsulation is suitable for precision systems (e.g., electronics or smart textiles), macroencapsulation is ideal for building and solar TES, and composite-encapsulation is designed for high-performance or industrial systems that necessitate robust thermal conductivity and structural integrity. Consequently, the subsequent section will delineate the specifics of each improvement strategy for encapsulating PCMs.

### 4.1. Microencapsulation

**Figure 12:**
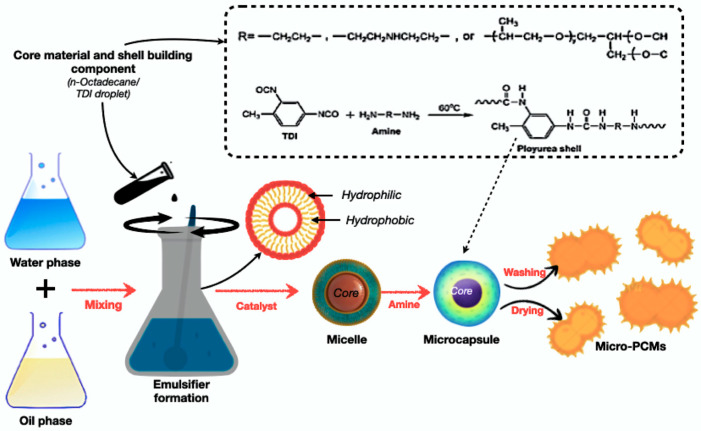
Schematic process of fabricating microencapsulated PCM using an oil-in-water emulsion method [156,157].

Microencapsulation coats PCM droplets or particles with protective shells, preventing leakage and improving dispersion [131,137]. It enables precise thermal control, but is costly and requires complex fabrication. Figure 12 illustrates the schematic representation of the microencapsulation process for PCMs using an oil-in-water emulsion method. The process involves emulsifying the PCM core in an aqueous continuous phase, followed by polymerisation or crosslinking to form stable microcapsules. This technique produces uniform, leakage-resistant particles that are suitable for thermal energy storage applications. Recent studies highlight the use of sustainable bio-based shell materials, such as lignin and chitosan, for improved compatibility and environmental performance [57,127].

However, microencapsulation is hampered by its high production cost and industrial complexity, which may impede scalability. Furthermore, shell fragility after repeated temperature cycling is a common problem, raising concerns regarding long-term durability. These restrictions highlight the necessity for hybrid shell designs that combine bio-based and synthetic components, balancing environmental performance and mechanical strength [36,57,143,158]. The thermal performance of the developed microcapsules was evaluated using Differential Scanning Calorimetry (DSC) in accordance with ASTM E793 and ISO 11357 [159,160]. These standards specify procedures for measuring the enthalpy of fusion and crystallisation, ensuring reliable determination of phase-change characteristics. The DSC analysis provided key data on melting temperature, latent heat, and thermal stability—essential parameters for assessing the efficiency of PCM-based thermal-energy-storage systems.

### 4.2. Macroencapsulation

**Figure 13:**
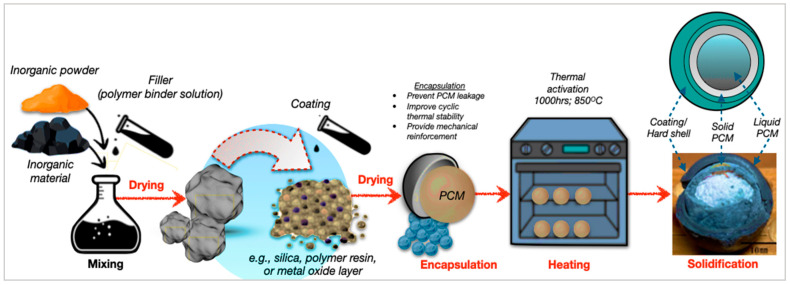
Schematic process of macroencapsulation of inorganic PCMs [145,157].

Otherwise, the macroencapsulation technique stores larger PCM volumes in capsules, tubes, or panels, offering high capacity and simple fabrication. However, it suffers from reduced heat-transfer efficiency, due to larger thermal gradients. Consequently, the process of the macroencapsulation of inorganic PCMs involves coating, encapsulation, and solidification steps. Figure 13 depicts the stepwise formation of macroencapsulated PCM composites, showing the preparation, thermal activation, and shell hardening stages. The approach, as demonstrated by Sivanathan et al. [144], effectively mitigates leakage and enhances thermal cycling stability and structural integrity. Compared to microencapsulation, macroencapsulation offers higher PCM capacity and simpler manufacturing, making it suitable for large-scale thermal regulation in building energy systems [156,157]. Moreover, Feizatidou et al. [158] have demonstrated that the nano/microencapsulation of PCMs using chemical techniques mitigates leakage in TES systems for buildings, which account for 40% of world energy consumption and 38% of emissions, hence improving structural integrity and efficiency. However, bio-based panels and fibre-reinforced shells provide substantial PCM-loading and protection; however, they have diminished heat transfer, due to bigger capsules and low surface area-to-volume ratios.

Furthermore, design constraints and the ongoing possibility of leakage remain obstacles in real-world deployment. Critically, while macroencapsulation provides cost-effective scaling, its efficiency trade-offs highlight the significance of including conductivity boosters or hybrid design features to compensate for decreased heat responsiveness. To assess the thermal efficiency and environmental durability of the macroencapsulated PCM composites, both thermal conductivity and hygrothermal exposure tests were conducted in accordance with ASTM E1225 and ISO 15927-3 standards [161,162]. The ASTM E1225 method enabled accurate determination of heat-transfer characteristics, while ISO 15927-3 guided the evaluation of façade exposure to driving rain and moisture loads. These analyses ensured comprehensive insight into the material’s thermal reliability and weather resistance for building-energy applications.

### 4.3. Composite-Encapsulation

**Figure 14:**
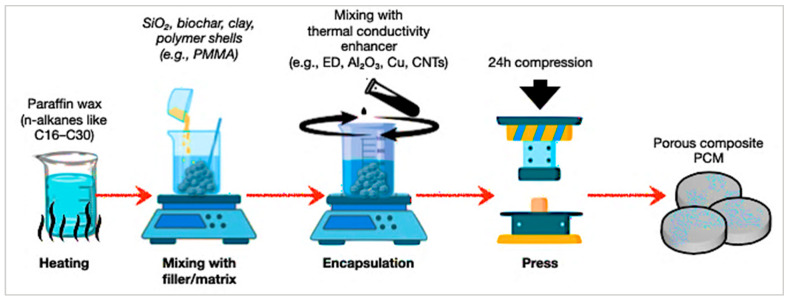
Schematic process of composite encapsulation for porous PCMs [145,151].

The composite-encapsulation technique embeds PCMs into supporting matrices such as polymers, aerogels, or porous scaffolds [131,137,138]. This enhances mechanical strength, stability, and thermal cycling performance, though PCM loading and conductivity are often limited. Figure 14 illustrates a composite encapsulation for porous PCMs. Paraffin wax is embedded into supporting fillers (e.g., SiO_2_, clays, or charcoal) and improved with conductivity agents such as expanded graphite, Al_2_O_3_, Cu, or CNTs. This technique physically integrates PCMs into solid matrices, decreasing leakage and greatly increasing thermal conductivity [146,147,148]. The thermal-transport characteristics of these composites were experimentally evaluated following ASTM E1461 and ISO 22007-2 standards [163,164]. The laser-flash method described in ASTM E1461 was used to determine thermal diffusivity, while the Transient Plane Heat Source (TPS) method defined in ISO 22007-2 enabled the measurement of both thermal conductivity and diffusivity. These standardised procedures ensured reliable quantification of heat-transfer enhancement attributed to conductive fillers and matrix architecture.

Meanwhile, bio-based composites (e.g., cellulose, lignin, starch, and proteins) are increasingly studied as eco-friendly encapsulation materials [131,149]. They improve biodegradability, mechanical stability, and long-term cycling performance [137,138]. Nonetheless, issues such as low thermal conductivity, mechanical degradation, and lifecycle emissions persist. The environmental compatibility of biochar and nanocellulose matrices corresponds with green material objectives, providing apparent advantages over petroleum-based shells. Shen et al. [150] discovered that nanocellulose improves sustainable PCMs for latent heat storage by utilising renewability and functionalisation to create composites such as slurries and capsules, thus addressing the limitations of PCMs.

Nonetheless, composite encapsulation frequently constrains PCM loading and matrix sensitivity, which may weaken energy density and repeatability in thermal storage applications. In addition, long-term thermal cycling stability remains an unresolved issue, particularly when PCMs are embedded in porous matrices that are prone to mechanical fatigue. Despite these constraints, composite encapsulation is regarded as one of the most promising strategies for achieving multifunctional PCMs, especially when combined with nano-engineered conductivity enhancers.

## 5. Future Directions and Research Opportunities

The extensive manufacturing of bio-based PCMs poses both technological and economic difficulties. The extraction and purification of natural feedstocks such as fatty acids, lignin, or cellulose derivatives necessitate multistep processes that elevate processing complexity and energy consumption. The expense of raw materials and encapsulating components is still elevated, relative to petroleum-based PCMs, hindering their extensive commercialisation. Nevertheless, novel green synthesis methods and the utilisation of inexpensive agricultural leftovers or industrial by-products are enhancing feasibility. Simultaneously, improvements in biopolymer modification and scalable encapsulation methods are decreasing processing expenses and improving performance uniformity, indicating that bio-based PCM systems are becoming competitive for industrial TES applications.

Moreover, subsequent research needed to concentrate on creating a systematic connection among material design strategies, scalable production methods, and application readiness evaluation. This integration will expedite the passage of bio-based PCM technology from laboratory invention to industrial application.

### 5.1. Advancements in Bio-Based Materials for PCM Encapsulation

Recent developments in bio-based materials have significantly enhanced the encapsulation performance of PCMs for TES. Ajdary et al. [152] demonstrated that plant-derived nanostructures, particularly nanocellulose-based hydrogels, foams, and aerogels, offer high mechanical strength and tuneable porosity, promoting effective PCM loading and improved thermal cycling stability. Likewise, the lignin-based composites investigated by Jyothibasu et al. [153] exhibited superior durability, due to electroactive quinone moieties within lignin’s aromatic matrix, which strengthen the interfacial bonding and resist degradation during repeated phase transitions. Furthermore, Atinafu et al. [154] reported that hybridised nanocellulose–lignin composites, reinforced with biochar, significantly enhance mechanical resilience, prevent leakage, and improve thermal conductivity through synergistic interactions between biopolymers and carbon. Collectively, these studies demonstrate the transformative potential of bio-derived hybrid systems as eco-friendly, structurally robust, and thermally efficient encapsulation matrices for next-generation TES applications.

Likewise, enhancing the thermal energy efficiency of bio-based PCM systems necessitates a multifaceted strategy that incorporates core material advancements, sophisticated encapsulation designs, and energy-retention enhancements. Table 4 summarises the approaches to the essential issues of low thermal conductivity, leakage, supercooling, and prolonged deterioration, facilitating the scalable and sustainable use of PCM technologies in the construction, textiles, electronic, and renewable energy sectors. Further improvements involve optimising bio-based PCMs for extreme environments. Suchorowiec et al. [155] observed that PCM–aerogel composites enhance form stability, conductivity, and flame retardancy for energy applications. Nanocellulose–lignin matrices with protective encapsulation withstand elevated temperatures and stress, while aerogels and graphene enhance conductivity and structural integrity. These hybrid materials ensure efficient energy storage and delivery across variable conditions. To fully realise their potential, future research must focus on the scalable production, cost-effective processing, and long-term performance of nano-enhanced bio-based PCMs. These innovations support high-performance, sustainable TES systems, and align with broader renewable energy goals [150,155,165].

### 5.2. Bio-Based PCM Core Innovations

Bio-based PCMs constitute a vital innovation avenue for enhancing the thermal energy efficiency of TES systems. In contrast to petroleum-derived paraffins, bio-based PCMs originate from renewable sources, including plant oils, fatty acids, biopolymers, and agricultural by-products, providing energy storage capabilities along with environmental advantages. Material advances emphasise mixed fatty acids (e.g., coconut oil and beeswax) that have adjustable melting points, suitable for various construction, food preservation, and HVAC applications [176,177,178].

**Figure 15:**
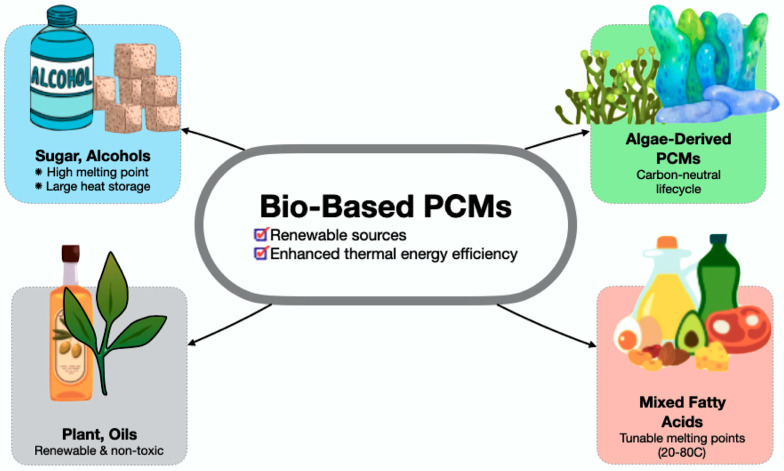
Bio-based PCM core innovations—renewable thermal energy efficiency [176,177,178].

Therefore, Figure 15 displays a categorisation of four classes of bio-based PCMs: sugar alcohols, algae-derived PCMs, plant oils, and mixed fatty acids, based on their energy density and environmental impact, highlighting material selection challenges for sustainable TES. These blends enable customised PCM cores that satisfy application-specific thermal demands. In addition, sugar alcohols, such as erythritol, exhibit elevated melting points and considerable latent heat capacity, whilst biopolymers like PLA, combined with waxes, provide opportunities for biodegradable and secure encapsulation. Brzęczek-Szafran et al. [177] propose a classification of biomass-derived PCMs (types 0–3) for sustainable composites, improving shape stability and conductivity while including green chemistry. Bio-based PCMs achieve a latent heat of 200–220 J/g through esterification, utilising waste for carbon-neutral energy production. Significantly, this places them in a competitive stance with traditional PCMs while providing superior environmental credentials. Thus, their carbon-neutral or carbon-negative lifecycles (e.g., algae-derived PCMs) reduce total greenhouse gas emissions, and offer a route to decarbonisation and an opportunity to develop secure, scalable, and adaptable TES materials for advanced energy systems.

### 5.3. Advanced Encapsulation

**Figure 16:**
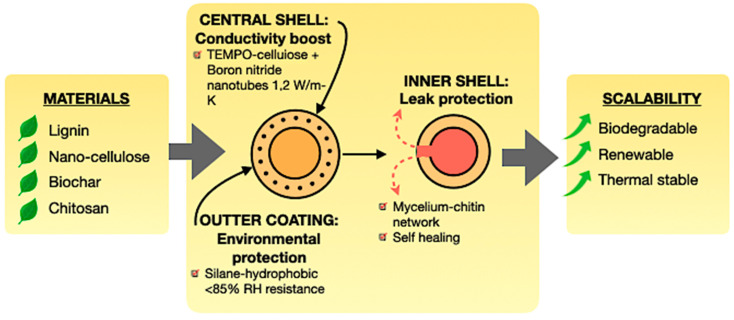
Advanced encapsulation strategies for bio-based PCMs [128,179,180].

Encapsulation converts PCMs from permeable, unstable substances into robust, high-performance composites. In addition to containment, contemporary encapsulation methods offer multifunctional enhancements, tackling leakage, conductivity, and environmental resilience [128,179,180]. Figure 16 shows the inner shell is engineered for leak protection, utilising mycelium–chitin networks that provide a distinctive self-healing ability, hence prolonging the PCM cycling life. The central shell, generally composed of TEMPO-cellulose, fortified with boron nitride nanotubes, elevates thermal conductivity, significantly enhancing heat absorption and dissipation rates. The external layer, typically a silane-hydrophobic coating, offers environmental protection, resisting humidity levels versus relative humidity and ensuring long-term stability in outdoor and construction applications [172,181,182,183,184].

Likewise, encapsulation techniques differ based on the magnitude of the application. Microencapsulation, utilising chitosan or cellulose nanocrystals, guarantees an extensive surface area and meticulous melting regulation, rendering it advantageous for electronic cooling applications. Macroencapsulation, such as wood aerogels or mycelium networks, offers scalable alternatives for building-integrated TES. Nanoencapsulation, such as liposomes or silica nanoparticles, facilitates the creation of ultra-thin shells with swift thermal responsiveness, which is ideal for biomedical and microelectronics applications, but also significantly improves conductivity, cycling stability, and environmental durability.

## 6. Future Bio-Based PCM Applications

The next generation of TES systems is influenced by the advancement of bio-based PCM-encapsulating substances that integrate elevated latent heat capacity with structural durability and sustainability. These materials mitigate the shortcomings of traditional PCMs, such as leakage, inadequate thermal conductivity, and degradation, through the incorporation of sophisticated encapsulation methods and multifunctional fillers. Table 5 delineates the thermal and structural characteristics of essential PCM encapsulation materials, emphasising their prospective applications in renewable energy systems: specifically, in solar thermal storage, sustainable architecture, intelligent textiles, and energy-efficient electronics. Moreover, subsequent research is needed to concentrate on creating a systematic connection among material design strategies, scalable production methods, and application readiness evaluation. This integration will expedite the passage of bio-based PCM technology from laboratory invention to industrial application.

### 6.1. Natural Fibre-PCM for EV Battery Thermal Management

**Figure 17:**
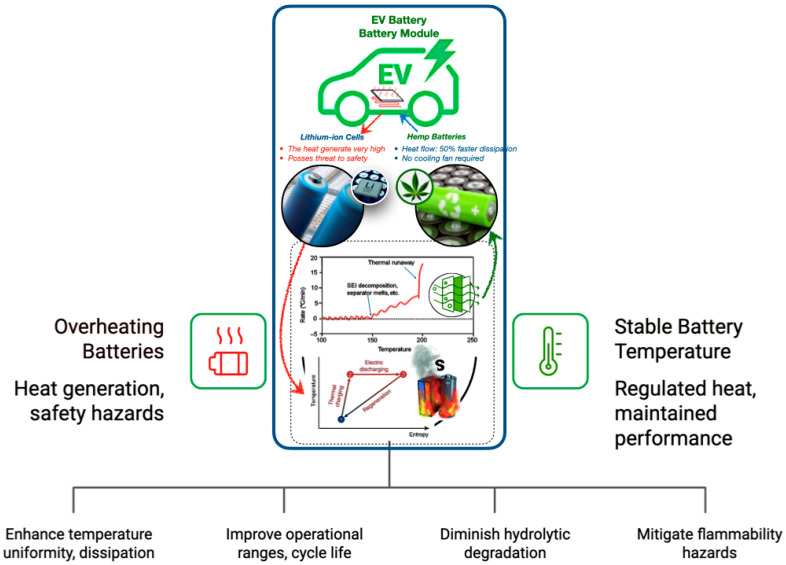
The revolutionary potential of bio-based encapsulated PCM systems with natural fibre-PCM for EV battery thermal management [57,208,209,211].

Bio-based fibre–PCM composites provide considerable benefits for thermal cycle stability in applications such as electric vehicle battery modules, providing enhanced temperature uniformity and accelerated heat dissipation, relative to traditional systems. These composites, fortified with natural fibres and including encapsulated PCMs, improve operational ranges and prolong cycle life, reducing Li-ion cell deterioration. Stability is enhanced by bio-based matrices that diminish hydrolytic degradation, while adjustable melting thresholds guarantee that PCM transitions stay within crucial battery safety limits [150,207,210,211]. Figure 17 demonstrates that traditional lithium-ion batteries experience significant heat generation and safety hazards during repeated charging and discharging cycles. Conversely, bio-based fibre–PCM composites (such as hemp or flax, reinforced with phase change materials) exhibit considerable benefits in regulating heat variations and maintaining performance throughout extended cycling [212,213].

Although these composites reduce the necessity for active cooling, flammability hazards are being mitigated by technologies such as lignin nanoparticle infusion. Notwithstanding certain constraints, including shell vulnerability under significant mechanical stress, the incorporation of bio-based encapsulated PCMs presents a lightweight, sustainable substitute for metallic cooling systems in electric vehicle batteries, with prospective uses in building-integrated TES and electronic cooling [57,208,209,211].

### 6.2. Wood–PCM Aerogel for Solar Window

Wood–PCM aerogels incorporated into solar windows provide both latent heat storage and photovoltaic generation, thus improving building envelopes beyond mere passive insulation [214,215,216,217,218,219,220,221,222,223,224,225]. The research conducted by Nazarit et al. [57] indicates that bio-based PCMs within wood matrices enhance thermal mass for sustainable buildings, attaining 15% solar efficiency, 100 J/g latent heat, and 88% transmittance, providing thermal cycle stability and optical clarity that are comparable to established standards. Furthermore, various studies validate the feasibility of aerogel composites for transparent TES, owing to their ultralight characteristics and adjustable conductivity, with wood-derived composites exhibiting structural durability [155,165,185].

**Figure 18:**
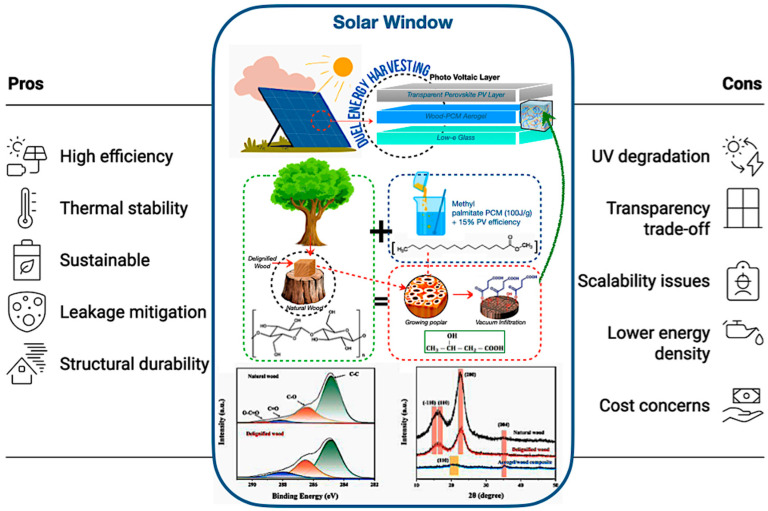
The revolutionary potential of bio-based encapsulated PCM systems of wood–PCM aerogel for solar window/panel [155,165,185].

As shown in Figure 18, the TEMPO-oxidation delignification method retains cellulose nanofibrils, ensuring optical clarity and addressing the shortcomings identified in previous cellulose-based encapsulation systems. Furthermore, the image illustrates that PCM microencapsulation of methyl palmitate contained within 50 μm cellulose nanocapsules mitigates leakage during successive solid–liquid transitions, an advancement corroborated by Lu et al. [226], who confirmed that microencapsulating palmityl palmitate in dicyclohexylmethane shells results in 84% encapsulation and an enthalpy of 218 J/g, maintaining over 90% efficiency after 500 cycles, and thereby improving the thermal regulation of epoxy resin without leakage. In addition, Figure 18 also illustrates the anisotropic thermal conductivity of the enhanced thermal stability by facilitating lateral heat transfer, while the wood matrix provides carbon sequestration advantages.

Despite ongoing challenges such as UV degradation, which can be alleviated by lignin nanoparticles but necessitates long-term validation, as well as trade-offs between PCM loading and optical transparency, bio-based aerogels present sustainable alternatives to inorganic PCMs. However, they exhibit lower energy density and scalability issues [113,128,226]. Thus, future research must focus on hybrid designs, standardised testing, and scalable processing to guarantee long-term stability and industrial acceptance, thereby establishing these windows as revolutionary for sustainable energy storage and smart building technologies.

### 6.3. Protein Adhesive PCM for Electronic Recycling

**Figure 19:**
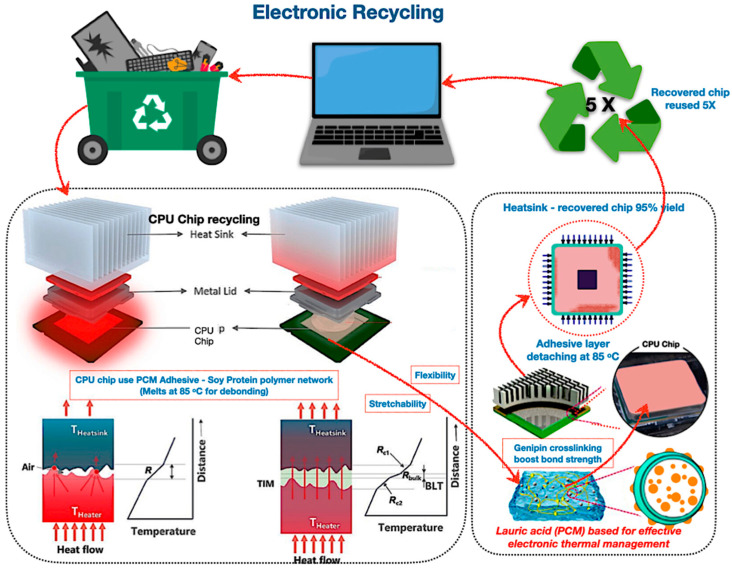
The revolutionary potential of bio-based encapsulated PCM systems of protein adhesive PCM for electronic recycling [214,215,216,217,218,219,220,221,222,223,224,225].

Protein-based adhesive PCMs offer an innovative solution for electronic heat management and component recycling, facilitating regulated debonding for optimal recovery rates. As illustrated in Figure 19, these bio-based materials possess reversible protein disulphide linkages, ensuring temperature cycle stability and self-healing properties. Further, bio-based adhesives demonstrate somewhat greater thermal resistance compared to grease-based thermal interface materials augmented with nanofillers. As non-toxic, sustainable alternatives, they facilitate circular flows; nonetheless, hybridisation and scalable crosslinking necessitate more exploration for performance enhancement.

In addition, protein-based adhesive PCMs are innovative soy protein adhesives, and PCMs such as lauric acid serve as thermal interface materials in the functioning of electronic devices. Szymanski and Paluch’s experiment [227] revealed that lauric acid PCMs in heat pipes reduce electronics’ cooling temperatures by up to 14.6% and store 25% more energy, surpassing soy wax, which achieves a 6.8% decrease and 12% storage capacity. Soy protein adhesives containing lauric acid facilitate controlled debonding at 85 °C for effective component recovery. In addition to thermal metrics, these protein adhesive PCMs provide environmentally advantageous characteristics, acting as non-toxic substitutes for epoxies and solder pastes by eliminating volatile organic compounds and heavy metals while establishing them as circular material recovery, making them recyclable and sustainable electronic materials.

### 6.4. PHA Biopolymer–PCM for Adsorbable Stents

**Figure 20:**
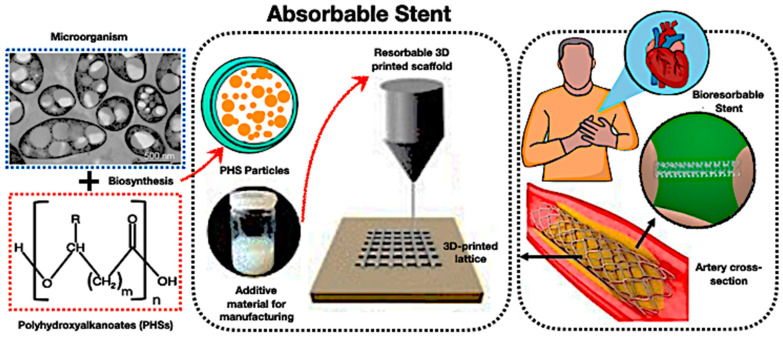
The revolutionary potential of bio-based encapsulated PCM systems of PHA biopolymer-PCM for absorbable stents [214,215,216,217,218,219,220,221,222,223,224,225].

Polyhydroxyalkanoates (PHAs) have emerged as viable options for bioresorbable scaffolds in cardiovascular applications, especially when integrated with PCMs. As depicted in Figure 20, PHAs can be biosynthesised into particles, fabricated into 3D-printed lattices, and utilised as resorbable stents that decompose after serving their structural purpose. The incorporation of encapsulated PCMs adds a novel aspect to thermal regulation, allowing stents to grow at a physiological temperature and provide regulated cooling effects to alleviate inflammation during plaque remodelling.

Moreover, Figure 20 demonstrates that PHA biopolymer–PCM stents represent the incorporation of thermal cycle stability, mechanical functionality, and biodegradability into a singular biomedical device. Despite the constraints of slow degradation rates, modest strength, and PCM stability, the system enhances scholarly discourse by demonstrating how bio-based TES composites might extend beyond conventional energy storage applications to meet biomedical and therapeutic requirements. Thus, these initiatives will elevate the function of PCM-loaded PHA scaffolds from experimental prototypes to clinically feasible, multifunctional solutions for next-generation absorbable stents.

Yuan et al. [228] outline encapsulation strategies (microencapsulation, electrospinning, and porous frameworks) to improve the reliability of PCMs, hence increasing medical applications in dressings, medication administration, cold chains, and bio-bone cement, while also delineating future hurdles. Furthermore, Wu et al. [229] emphasise that shape memory scaffolds outperform metallic implants through adaptive recovery, facilitating minimally invasive bone engineering, cardiovascular stenting, and tissue regeneration; PHA–PCM composites provide thermal modulation and biodegradability, addressing significant clinical challenges. Consequently, encapsulation strategies improve the reliability of PCM for medical applications, such as dressings and drug delivery, whereas shape memory scaffolds incorporating PHA–PCM composites provide adaptive, biodegradable substitutes for metallic implants in minimally invasive tissue engineering and cardiovascular therapies.

## 7. Conclusions

TES using PCMs is a promising solution for energy efficiency, but issues like low thermal conductivity, leakage, and phase instability reduce performance. This review highlights previous studies on bio-based composite encapsulation as a sustainable way to improve PCM reliability, thermal conductivity, and structural stability. Materials such as lignin, nanocellulose, and biochar enhance heat transfer and reduce leakage, while hybrid nanocomposites, like graphene–biochar blends, create efficient thermal pathways. Bio-based systems also offer environmental benefits, including biodegradability and reduced carbon emissions. Applications in solar heating, wind power stabilisation, and building insulation show improved energy retention and thermal regulation, positioning them as viable alternatives to petroleum-based solutions.

However, challenges like scalability and cost remain. Most advances are still at the lab scale, with limited industrial applications. Future efforts should focus on developing low-cost methods, such as spray drying and 3D printing, to enable large-scale production. Long-term durability, especially under extreme conditions, also needs more investigation. Techniques like nano-reinforcement and crosslinking could help improve resistance to oxidation, moisture, and degradation. There is also potential for developing multi-functional bio-based PCMs with self-healing, shape memory, or conductive properties. Hybrid systems combining biochar with aerogels or metal–organic frameworks could further boost efficiency. Integrating computational modelling and AI-driven design may also optimise PCM performance for specific applications. In summary, bio-based encapsulated PCMs offer a sustainable path forward for TES. Advancing these materials through interdisciplinary research could lead to more efficient, scalable, and eco-friendly energy storage solutions.

## Figures and Tables

**Table 2:** Comparison of thermal conductivity and latent heat storage capacity in bio-based vs. traditional PCM encapsulation.

Encapsulation Material	Thermal Conductivity (W/m·K)	Latent Heat Storage Capacity (J/g)	Advantages	Disadvantages	References
Starch (Bio-Based)	0.16–0.25	100–160	Renewable, biodegradable Enhanced by nanofillers or crosslinkers	Low moisture resistance Moderate thermal stability	[46,47,48,49,50,51]
Chitosan (Bio-Based)	0.20–0.30	120–180	Antimicrobial, biodegradable Good film-forming ability	Sensitive to pH and moisture Moderate mechanical strength	[52,53,54,55,56]
Lignocellulosic (Bio-Based)	0.20–0.42	150–210	High mechanical strength Abundant and low cost Good compatibility with PCMs	Brittleness without plasticizers Requires surface modification	[57,58,59,60,61]
Silica (inorganic)	1.4–1.6	200–250	Excellent thermal stability High mechanical strength Chemically inert	Brittle, prone to cracking Non-biodegradable	[62,63,64,65,66]
Polystyrene (inorganic)	0.1–0.13	180–240	Good processability Lightweight and low cost	Low thermal conductivity Derived from petroleum sources	[67,68,69,70]
Polymethyl methacrylate (PMMA) (inorganic)	0.18–0.25	170–230	Transparent, good flexibility Excellent encapsulation efficiency	Low thermal conductivity Sensitive to UV degradation	[71,72,73,74,75]

**Table 3:** Comparison of PCM encapsulation enhancement techniques.

Encapsulation Technique	Structural Characteristics	Advantages	Disadvantages	Applications	ASTM/ISO Standard	References
Microencapsulation	PCM particles (1–1000 µm) coated with polymer or bio-based shells (e.g., chitosan, lignin).	Prevents leakage. Improves heat transfer. Enables uniform melting/solidifying.	High cost. Complex production. Shell may crack after many cycles.	Electronics. Smart textiles. Building panels. Solar TES.	ASTM E793/ISO 11357: DSC phase change test. ASTM D3849: Microcapsule size/morphology.	[132,136,140,143,144,145,146]
Macroencapsulation	PCM sealed in large modules (tubes, panels, or spheres).	Simple and low-cost. Suitable for large-scale TES. Easy handling and reuse.	Lower heat transfer. Leakage risk at joints. Bulky for small systems.	Building HVAC. Water heater. Solar panels.	ISO 15927-3: Thermal performance of materials. ASTM E1225: Thermal conductivity.	[147,148,149,150]
Composite encapsulation	PCM mixed with porous or conductive frameworks (graphite, silica, metal foam).	High thermal conductivity. Leak-free and stable. Shape-stable PCM.	Reduce latent heat. Complex processing. Expensive materials.	Battery cooling. Electronic. High-temperature TES.	ASTM E1461/ISO 22007-2: Thermal diffusivity and conductivity.	[151,152,153,154,155]

**Table 4:** Thermal performance of nano-enhanced bio-based PCM composites.

Composite Type	Nano-Additive	PCM Leakage Prevention	Application Scope	References
Nanocellulose–PCM	▯ Boron Nitride Nanotubes (BNNTs) Cellulose Nanocrystals (CNCs)	▯ Microfibril network reduces leakage Swelling-induced self-sealing	Building insulation Wearable textiles	[154,166,167,168]
Biochar–PCM	▯ Graphene-coated biochar Carbon nanotubes (CNTs)	▯ Hierarchical pores trap PCM 0% leakage at 80 °C tilt	Solar thermal storage Industrial waste heat recovery	[169,170,171,172]
Graphene–PCM	▯ Graphene oxide (GO) Reduced graphene oxide (rGO)	▯ Impermeable graphene layers 100% leakage prevention up to 120 °C	Electronics cooling High-power battery thermal management	[168,173,174,175]

**Table 5:** Thermal and structural properties of PCM encapsulation materials with future application.

Material	Properties	Thermal Conductivity (W/m·K)	Latent Heat Capacity (J/g)	Cycle Stability (Cycles)	Future Applications	References
Nanocellulose (hydrogels)	Porous Anisotropic structure Self-healing ability	0.12–0.25	160–180	3000–5000	4D-printed smart textiles Implantable thermal therapy devices	[150,152,185,186,187]
Lignin-based polymers	UV-absorbing Radical scavenging	0.18–0.30	140–170	8000–12,000	Solar farm thermal buffers Recyclable packaging	[153,188,189,190,191]
Biochar composites	Hierarchical pores (10 nm–5 µm) 500 m^2^/g surface area Electrically conductive	0.30–1.50	120–150	20,000+	EV battery thermal runaway Industrial waste heat recovery sponges	[154,192,193,194,195]
Nanocellulose–lignin	Synergistic H-bonding Vibration damping Moisture-resistant	0.025–0.045	60–190	15,000–18,000	Aerospace cabin panels Shock-resistant military gear	[143,150,152,153,186]
Wood-based hydrogels	Native cellulose alignment Microfluidic channels Carbon negative	0.18–0.90	100–180	5000–7000	Transparent wood smart windows Heat transfer building materials	[152,196,197,198,199,200]
Conventional polymer PCM	Consistent quality Wide availability Petrochemical base	0.30–2.90	120–210	10,000–15,000	HVAC systems Low-cost consumer goods	[21,201,202,203,204,205,206]
Paraffin in microcapsules	High shell (enthalpy) Hermetic sealing	0.60–4.00	80–200	50,000+	Thermal switches Nuclear reactor passive cooling systems	[178,207,208,209]

## Data Availability

The original contributions presented in this study are included in the article. Further inquiries can be directed to the corresponding author.

## References

[1] Alva G., Lin Y., Fang G. (2018). An overview of thermal energy storage systems. Energy.

[2] Pelay U., Luo L., Fan Y., Stitou D., Rood M. (2017). Thermal energy storage systems for concentrated solar power plants. Renew. Sustain. Energy Rev..

[3] Akinyele D.O., Rayudu R.K. (2014). Review of energy storage technologies for sustainable power networks. Sustain. Energy Technol. Assessments.

[4] El Majd A., Sair S., Ait Ousaleh H., Berardi U., Moulakhnif K., Belouaggadia N., Younsi Z., El Bouari A. (2024). Advancing PCM research in building efficiency: A comprehensive investigation into PCM selection and critical integration strategies. J. Build. Eng..

[5] Rashid F.L., Al-Obaidi M.A., Dhaidan N.S., Hussein A.K., Ali B., Ben Hamida M.B., Younis O. (2023). Bio-based phase change materials for thermal energy storage and release: A review. J. Energy Storage.

[6] Baylis C., Cruickshank C.A. (2023). Review of bio-based phase change materials as passive thermal storage in buildings. Renew. Sustain. Energy Rev..

[7] Liu S., Wu H., Du Y., Lu X., Qu J. (2021). Shape-stable composite phase change materials encapsulated by bio-based balsa wood for thermal energy storage. Sol. Energy Mater. Sol. Cells.

[8] Soares N., Costa J.J., Gaspar A.R., Santos P. (2013). Review of passive PCM latent heat thermal energy storage systems towards buildings’ energy efficiency. Energy Build..

[9] Zhang K., Hu C., Huang H., Li B., Huang C., Wang S. (2024). Achieving efficient energy utilization by PCM in the food supply chain: Encapsulation technologies, current applications, and future prospects. J. Energy Storage.

[10] Wang X., Li W., Luo Z., Wang K., Shah S.P. (2022). A critical review on phase change materials (PCM) for sustainable and energy efficient building: Design, characteristic, performance and application. Energy Build..

[11] Panwar N.L., Kaushik S.C., Kothari S. (2011). Role of renewable energy sources in environmental protection: A review. Renew. Sustain. Energy Rev..

[12] Rathod M.K., Banerjee J. (2013). Thermal stability of phase change materials used in latent heat energy storage systems: A review. Renew. Sustain. Energy Rev..

[13] Bošnjak Hordov J., Nižetić S., Jurčević M., Čoko D., Ćosić M., Jakić M., Arıcı M. (2024). Review of organic and inorganic waste-based phase change composites in latent thermal energy storage: Thermal properties and applications. Energy.

[14] Singh P., Sharma R.K., Ansu A.K., Goyal R., Sarı A., Tyagi V.V. (2021). A comprehensive review on development of eutectic organic phase change materials and their composites for low and medium range thermal energy storage applications. Sol. Energy Mater. Sol. Cells.

[15] Garivalis A.I., Rossi D., Seggiani M., Testi D. (2024). Beyond water: Physical and heat transfer properties of phase change slurries for thermal energy storage. Cell Reports Phys. Sci..

[16] Jagadeeswara Reddy V., Fairusham Ghazali M., Kumarasamy S. (2024). Innovations in phase change materials for diverse industrial applications: A comprehensive review. Results Chem..

[17] Liu Y., Zheng R., Li J. (2022). High latent heat phase change materials (PCMs) with low melting temperature for thermal management and storage of electronic devices and power batteries: Critical review. Renew. Sustain. Energy Rev..

[18] Eanest Jebasingh B., Valan Arasu A. (2020). A comprehensive review on latent heat and thermal conductivity of nanoparticle dispersed phase change material for low-temperature applications. Energy Storage Mater..

[19] Reddy K.S., Mudgal V., Mallick T.K. (2018). Review of latent heat thermal energy storage for improved material stability and effective load management. J. Energy Storage.

[20] Jouhara H., Żabnieńska-Góra A., Khordehgah N., Ahmad D., Lipinski T. (2020). Latent thermal energy storage technologies and applications: A review. Int. J. Thermofluids.

[21] Zhang N., Yuan Y., Cao X., Du Y., Zhang Z., Gui Y. (2018). Latent Heat Thermal Energy Storage Systems with Solid–Liquid Phase Change Materials: A Review. Adv. Eng. Mater..

[22] Aftab W., Usman A., Shi J., Yuan K., Qin M., Zou R. (2021). Phase change material-integrated latent heat storage systems for sustainable energy solutions. Energy Environ. Sci..

[23] Li Z.-R., Hu N., Fan L.-W. (2023). Nanocomposite phase change materials for high-performance thermal energy storage: A critical review. Energy Storage Mater..

[24] Kurnia J.C., Haryoko L.A.F., Taufiqurrahman I., Chen L., Jiang L., Sasmito A.P. (2022). Optimization of an innovative hybrid thermal energy storage with phase change material (PCM) wall insulator utilizing Taguchi method. J. Energy Storage.

[25] Mika Ł., Radomska E., Sztekler K., Gołdasz A., Zima W. (2025). Review of Selected PCMs and Their Applications in the Industry and Energy Sector. Energies.

[26] Zhao Y., Zhang X., Hua W. (2021). Review of preparation technologies of organic composite phase change materials in energy storage. J. Mol. Liq..

[27] Irfan Lone M., Jilte R. (2021). A review on phase change materials for different applications. Mater. Today Proc..

[28] Yang G., Yim Y.-J., Lee J.W., Heo Y.-J., Park S.-J. (2019). Carbon-Filled Organic Phase-Change Materials for Thermal Energy Storage: A Review. Molecules.

[29] Mehta P., Patel V., Kumar S., Sharma V., Tejani G.G., Santhosh A.J. (2025). Performance assessment of thermal energy storage system for solar thermal applications. Sci. Rep..

[30] Zhang H., Xu C., Fang G. (2022). Encapsulation of inorganic phase change thermal storage materials and its effect on thermophysical properties: A review. Sol. Energy Mater. Sol. Cells.

[31] Rao G.A.P., Valaboju S.K., Babu A., Saurav G.V. (2025). Comparison between organic and inorganic PCMs providing effective battery thermal management-A Machine Learning Approach. Futur. Batter..

[32] Naresh R., Parameshwaran R., Vinayaka Ram V. (2020). Bio-based phase-change materials. Bio-Based Materials and Biotechnologies for Eco-Efficient Construction.

[33] Mehrizi A.A., Karimi-Maleh H., Naddafi M., Karimi F. (2023). Application of bio-based phase change materials for effective heat management. J. Energy Storage.

[34] Boussaba L., Lefebvre G., Makhlouf S., Grados A., Royon L. (2021). Investigation and properties of a novel composite bio-PCM to reduce summer energy consumptions in buildings of hot and dry climates. Sol. Energy.

[35] Pundienė I., Pranckevičienė J., Bumanis G., Šinka M., Bajare D. (2025). Experimental investigation of novel bio-composite with integrated phase change materials (PCM) for enhanced energy saving in buildings. Ind. Crops Prod..

[36] Pielichowska K., Nowicka-Dunal K., Pielichowski K. (2024). Bio-Based Polymers for Environmentally Friendly Phase Change Materials. Polymers.

[37] Sun M., Liu T., Sha H., Li M., Liu T., Wang X., Chen G., Wang J., Jiang D. (2023). A review on thermal energy storage with eutectic phase change materials: Fundamentals and applications. J. Energy Storage.

[38] Huang J., Lu S., Kong X., Liu S., Li Y. (2013). Form-Stable Phase Change Materials Based on Eutectic Mixture of Tetradecanol and Fatty Acids for Building Energy Storage: Preparation and Performance Analysis. Materials.

[39] Parsamanesh M., Shekarriz S., Montazer M. (2025). Enhanced thermal stability of eutectic PCMs via microencapsulation: Inverse emulsion polymerization with silica shells. Therm. Sci. Eng. Prog..

[40] Aiduang W., Chanthaluck A., Kumla J., Jatuwong K., Srinuanpan S., Waroonkun T., Oranratmanee R., Lumyong S., Suwannarach N. (2022). Amazing Fungi for Eco-Friendly Composite Materials: A Comprehensive Review. J. Fungi.

[41] Hussin M.H., Abd Latif N.H., Hamidon T.S., Idris N.N., Hashim R., Appaturi J.N., Brosse N., Ziegler-Devin I., Chrusiel L., Fatriasari W. (2022). Latest advancements in high-performance bio-based wood adhesives: A critical review. J. Mater. Res. Technol..

[42] Avella M., Buzarovska A., Errico M.E., Gentile G., Grozdanov A. (2009). Eco-Challenges of Bio-Based Polymer Composites. Materials.

[43] Raydan N.D.V., Leroyer L., Charrier B., Robles E. (2021). Recent Advances on the Development of Protein-Based Adhesives for Wood Composite Materials—A Review. Molecules.

[44] Maulana M.I., Lubis M.A.R., Febrianto F., Hua L.S., Iswanto A.H., Antov P., Kristak L., Mardawati E., Sari R.K., Zaini L.H. (2022). Environmentally Friendly Starch-Based Adhesives for Bonding High-Performance Wood Composites: A Review. Forests.

[45] Chang B.P., Mohanty A.K., Misra M. (2020). Studies on durability of sustainable biobased composites: A review. RSC Adv..

[46] Kong Q., Mu H., Han Y., Wu W., Tong C., Fang X., Liu R., Chen H., Gao H. (2021). Biodegradable phase change materials with high latent heat: Preparation and application on Lentinus edodes storage. Food Chem..

[47] Öztürk G., Temiz A., Hekimoğlu G., Aslan M., Demirel G.K., Erdeyer Ö.N., Sarı A., Gencel O., Subaşı S. (2024). Microencapsulated phase change material/wood fiber-starch composite as novel bio-based energy storage material for buildings. J. Energy Storage.

[48] Gadhave P., Pathan F., Kore S., Prabhune C. (2022). Comprehensive review of phase change material based latent heat thermal energy storage system. Int. J. Ambient Energy.

[49] Wang T., Zhao S., Liu S., Li J., Xin Y., Lu Q., Chen H., Liang C. (2022). Effect of porous carbon on thermal and physical properties of composite pure alkane/expanded vermiculite phase change energy storage materials. J. Energy Storage.

[50] Shi T., Zhang X., Qiao J., Wu X., Chen G., Leng G., Lin F., Min X., Huang Z. (2021). Preparation and characterization of composite phase change materials based on paraffin and carbon foams derived from starch. Polymer.

[51] Wang Q., Tan X., Nian H., Wang X., Xue C., Zhao Y., Wang Z., Zhou Y. (2024). CH_3_COONa·3H_2_O/flaky modified vermiculite composite phase change materials enhanced phase change behavior and thermal storage through curing lotus root starch modification. J. Energy Storage.

[52] El Majd A., Sair S., Ousaleh H.A., Moulakhnif K., Younsi Z., Belouaggadia N., El Bouari A. (2024). Advanced gypsum/PCM composites incorporating biopolymer-encapsulated phase change materials for enhanced thermal management in buildings. Constr. Build. Mater..

[53] Atinafu D.G., Yang S., Yun B.Y., Kang Y., Kim S. (2022). Use of biochar co-mediated chitosan mesopores to encapsulate alkane and improve thermal properties. Environ. Res..

[54] Xu X., Yin S., Zhai X., Wu Z., Wang J., Ma J., Peng X., Peng H. (2024). Chitosan-based bilayer shell phase change nano-capsules with excellent anti-permeability for thermal regulation dressings. J. Energy Storage.

[55] Chen Y., Yang M., Song Y., Sun C., Tan H., Zheng D., Zhang Y. (2025). Leak-proof wood-based phase change materials composites utilizing chitosan encapsulation technology. J. Energy Storage.

[56] Yin G.-Z., Yang X.-M., López A.M., Wang M.-T., Ye W., Xu B., Wang D.-Y. (2022). Sodium alginate and Chitosan aided design of form-stable Polyrotaxane based phase change materials with ultra-high latent heat. Int. J. Biol. Macromol..

[57] Nazari M., Jebrane M., Terziev N. (2020). Bio-Based Phase Change Materials Incorporated in Lignocellulose Matrix for Energy Storage in Buildings—A Review. Energies.

[58] Sun M., Feng Y., Di H., Lin L. (2024). Evaluation into the effect of lignocellulosic biochar on the thermal properties of shape stable composite phase change materials. Ind. Crops Prod..

[59] Can A. (2023). Preparation, characterization, and thermal properties of microencapsulated palmitic acid with ethyl cellulose shell as phase change material impregnated wood. J. Energy Storage.

[60] Hu X., Kankkunen A., Seppälä A., Yazdani McCord M.R. (2024). Scaling up hybrid insulation: Integration of lignocellulose and phase change materials for sustainable thermal management. Mater. Today Commun..

[61] Nazari M., Jebrane M., Terziev N. (2022). Solid wood impregnated with a bio-based phase change material for low temperature energy storage in building application. J. Therm. Anal. Calorim..

[62] Mitran R.-A., Ioniţǎ S., Lincu D., Berger D., Matei C. (2021). A Review of Composite Phase Change Materials Based on Porous Silica Nanomaterials for Latent Heat Storage Applications. Molecules.

[63] Ismail A., Zhou J., Aday A., Davidoff I., Odukomaiya A., Wang J. (2023). Microencapsulation of bio-based phase change materials with silica coated inorganic shell for thermal energy storage. J. Build. Eng..

[64] Cao F., Li Z., Zhang Y., Wang X., Zhu L., Zhang S., Tang B. (2024). Silica-based aerogels encapsulate organic/inorganic composite phase change materials for building thermal management. J. Energy Storage.

[65] Zhang H., Wang X., Wu D. (2010). Silica encapsulation of n-octadecane via sol–gel process: A novel microencapsulated phase-change material with enhanced thermal conductivity and performance. J. Colloid Interface Sci..

[66] Tan N., Ning Y.-H., Hu P., Feng Y., Li Q., Lin C.-H., Cao Z., Zhang Y.-F., Zeng J.-L. (2022). Silica-confined composite form-stable phase change materials: A review. J. Therm. Anal. Calorim..

[67] Sarı A., Alkan C., Altıntaş A. (2014). Preparation, characterization and latent heat thermal energy storage properties of micro-nanoencapsulated fatty acids by polystyrene shell. Appl. Therm. Eng..

[68] Sarı A., Alkan C., Kahraman Döğüşcü D., Biçer A. (2014). Micro/nano-encapsulated n-heptadecane with polystyrene shell for latent heat thermal energy storage. Sol. Energy Mater. Sol. Cells.

[69] Sarı A., Alkan C., Döğüşcü D.K., Kızıl Ç. (2015). Micro/nano encapsulated n-tetracosane and n-octadecane eutectic mixture with polystyrene shell for low-temperature latent heat thermal energy storage applications. Sol. Energy.

[70] Sami S., Sadrameli S.M., Etesami N. (2018). Thermal properties optimization of microencapsulated a renewable and non-toxic phase change material with a polystyrene shell for thermal energy storage systems. Appl. Therm. Eng..

[71] Sarı A., Alkan C., Bilgin C. (2014). Micro/nano encapsulation of some paraffin eutectic mixtures with poly(methyl methacrylate) shell: Preparation, characterization and latent heat thermal energy storage properties. Appl. Energy.

[72] Alkan C., Sari A. (2008). Fatty acid/poly(methyl methacrylate) (PMMA) blends as form-stable phase change materials for latent heat thermal energy storage. Sol. Energy.

[73] Sarı A., Alkan C., Biçer A., Altuntaş A., Bilgin C. (2014). Micro/nanoencapsulated n-nonadecane with poly(methyl methacrylate) shell for thermal energy storage. Energy Convers. Manag..

[74] Liu X., Fleischer A., Feng G. (2021). Nanoencapsulated Lauric Acid with a Poly(methyl methacrylate) Shell for Thermal Energy Storage with Optimum Capacity and Reliability. ACS Appl. Polym. Mater..

[75] Yang Y., Ye X., Luo J., Song G., Liu Y., Tang G. (2015). Polymethyl methacrylate based phase change microencapsulation for solar energy storage with silicon nitride. Sol. Energy.

[76] Yadav A., Pandey A.K., Samykano M., Kalidasan B., Said Z. (2024). A review of organic phase change materials and their adaptation for thermal energy storage. Int. Mater. Rev..

[77] Coetzee D., Venkataraman M., Militky J., Petru M. (2020). Influence of nanoparticles on thermal and electrical conductivity of composites. Polymers.

[78] Zhou Y., Wu S., Ma Y., Zhang H., Zeng X., Wu F., Liu F., Ryu J.E., Guo Z. (2020). Recent Advances in Organic/Composite Phase Change Materials for Energy Storage. ES Energy Environ..

[79] Attias N., Danai O., Tarazi E., Pereman I., Grobman Y.J. (2019). Implementing bio-design tools to develop mycelium-based products. Des. J..

[80] Das D., Bordoloi U., Muigai H.H., Kalita P. (2020). A novel form stable PCM based bio composite material for solar thermal energy storage applications. J. Energy Storage.

[81] Lee W., Lee J., Yang W., Kim J. (2023). Fabrication of Biobased Advanced Phase Change Material and Multifunctional Composites for Efficient Thermal Management. ACS Sustain. Chem. Eng..

[82] Zhang Q., Liu J., Zhang J., Lin L., Shi J. (2022). A Review of Composite Phase Change Materials Based on Biomass Materials. Polymers.

[83] Ahmed T., Toki G.F.I., Mia R., Faridul Hasan K.M., Alpár T. (2024). Mechanical and Thermal Properties of Plant/Plant Fiber Based Woven Fabric Hybrid Composites. Innovations in Woven and Non-Woven Fabrics Based Laminated Composites. Composites Science and Technology.

[84] Owen M.M., Achukwu E.O., Romli A.Z., Md Akil H. (2023). Recent advances on improving the mechanical and thermal properties of kenaf fibers/engineering thermoplastic composites using novel coating techniques: A review. Compos. Interfaces.

[85] Wang X., Ma B., Wei K., Zhang W. (2021). Thermal stability and mechanical properties of epoxy resin/microcapsule composite phase change materials. Constr. Build. Mater..

[86] Rashid F.L., Al-Obaidi M.A., Hatem W.A., Almuhanna R.R.A., Abdul Redha Z.A., Al Maimuri N.M.L., Dulaimi A. (2025). Assessing the Effect of Organic, Inorganic, and Hybrid Phase Change Materials on Thermal Regulation and Energy Efficiency in Asphalt Pavements—A Review. Processes.

[87] Zhao F., Yuan W., Chen H., Fu H., Li Z., Xiao J., Feng Y. (2025). Advances in Organic Porous Polymeric-Supported Photothermal Phase Change Materials. Carbon Energy.

[88] Yang M., Zhong H., Li T., Wu B., Wang Z., Sun D. (2023). Phase Change Material Enhanced Radiative Cooler for Temperature-Adaptive Thermal Regulation. ACS Nano.

[89] Sun X., Zhang Y., Xie K., Medina M.A. (2022). A parametric study on the thermal response of a building wall with a phase change material (PCM) layer for passive space cooling. J. Energy Storage.

[90] Cuce P.M. (2025). Sustainable Insulation Technologies for Low-Carbon Buildings: From Past to Present. Sustainability.

[91] Carlucci F., Campagna L.M., Fiorito F. (2024). Responsive Envelope Technologies. Responsive Envelopes and Climate Change. Digital Innovations in Architecture, Engineering and Construction.

[92] Tao P., Mccafferty D.J. (2018). Bioinspired Thermal Insulation and Storage Materials. Bioinspired Engineering of Thermal Materials.

[93] Kalidasan B., Pandey A.K. (2025). Next generation phase change materials: State-of-the-art towards sustainable future. Prog. Mater. Sci..

[94] McBane J.E., Vulesevic B., Padavan D.T., McEwan K.A., Korbutt G.S., Suuronen E.J. (2013). Evaluation of a Collagen-Chitosan Hydrogel for Potential Use as a Pro-Angiogenic Site for Islet Transplantation. PLoS ONE.

[95] Keplinger T., Wang X., Burgert I. (2019). Nanofibrillated cellulose composites and wood derived scaffolds for functional materials. J. Mater. Chem. A.

[96] Mohseni A., Vieira F.R., Pecchia J.A., Gürsoy B. (2023). Three-Dimensional Printing of Living Mycelium-Based Composites: Material Compositions, Workflows, and Ways to Mitigate Contamination. Biomimetics.

[97] Liu Z., Sheng Z., Bao Y., Cheng Q., Wang P.-X., Liu Z., Zhang X. (2023). Ionic Liquid Directed Spinning of Cellulose Aerogel Fibers with Superb Toughness for Weaved Thermal Insulation and Transient Impact Protection. ACS Nano.

[98] Chen G., Li T., Chen C., Wang C., Liu Y., Kong W., Liu D., Jiang B., He S., Kuang Y. (2019). A Highly Conductive Cationic Wood Membrane. Adv. Funct. Mater..

[99] Islam M.N., Rahman F., Das A.K., Hiziroglu S. (2022). An overview of different types and potential of bio-based adhesives used for wood products. Int. J. Adhes. Adhes..

[100] Sayfutdinova A.R., Bardina K.A., Cherednichenko K.A., Vinokurov V.A., Voronin D.V. (2023). Effect of Fungal Mycellium/Eicosane Composites on Thermal Energy Storage and Release in Gypsum Plaster. Chem. Technol. Fuels Oils.

[101] Sayfutdinova A.R., Zaytseva N.E., Karsukova A.D., Cherednichenko K.A., Stoporev A.S., Vinokurov V.A., Voronin D.V. (2024). Case Study of Fungal Mycelium/Eicosane Composite as an Energy Storage Additive for Gypsum Plaster in a Model Experiment. Chem. Technol. Fuels Oils.

[102] Lv S., Liang S., Zuo J., Zhang S., Wang J., Wei D. (2023). Lignin-based anti-UV functional materials: Recent advances in preparation and application. Iran. Polym. J..

[103] Kaur R., Bhardwaj S.K., Chandna S., Kim K.H., Bhaumik J. (2021). Lignin-based metal oxide nanocomposites for UV protection applications: A review. J. Clean. Prod..

[104] Wang M., Shao C., Zhou S., Yang J., Xu F. (2017). Preparation of carbon aerogels from TEMPO-oxidized cellulose nanofibers for organic solvents absorption. RSC Adv..

[105] Le W.T., Kankkunen A., Rojas O.J., Yazdani M.R. (2023). Leakage-free porous cellulose-based phase change cryogels for sound and thermal insulation. Sol. Energy Mater. Sol. Cells.

[106] Wang G., Tang Z., Gao Y., Liu P., Li Y., Li A., Chen X. (2023). Phase Change Thermal Storage Materials for Interdisciplinary Applications. Chem. Rev..

[107] Kontturi K.S., Lee K.-Y., Jones M.P., Sampson W.W., Bismarck A., Kontturi E. (2021). Influence of biological origin on the tensile properties of cellulose nanopapers. Cellulose.

[108] Li J., Chen S., Li X., Zhang J., Nawaz H., Xu Y., Kong F., Xu F. (2023). Anisotropic cellulose nanofibril aerogels fabricated by directional stabilization and ambient drying for efficient solar evaporation. Chem. Eng. J..

[109] Rossetti A., Paciaroni A., Rossi B., Bottari C., Comez L., Corezzi S., Melone L., Almásy L., Punta C., Fiorati A. (2023). TEMPO-oxidized cellulose nanofibril/polyvalent cations hydrogels: A multifaceted view of network interactions and inner structure. Cellulose.

[110] Isogai A., Hänninen T., Fujisawa S., Saito T. (2018). Review: Catalytic oxidation of cellulose with nitroxyl radicals under aqueous conditions. Prog. Polym. Sci..

[111] Wang S., Yu L., Jia X., Zhang L., Liu H., Gao E., Chen C. (2024). Cellulose nanofibril-guided orienting response of supramolecular network enables superstretchable, robust, and antifatigue hydrogel. Innov. Mater..

[112] Yazdani M.R., Ajdary R., Kankkunen A., Rojas O.J., Seppälä A. (2021). Cellulose Nanofibrils Endow Phase-Change Polyethylene Glycol with Form Control and Solid-to-gel Transition for Thermal Energy Storage. ACS Appl. Mater. Interfaces.

[113] Al-Shannaq R., Farid M.M., Ikutegbe C.A. (2022). Methods for the Synthesis of Phase Change Material Microcapsules with Enhanced Thermophysical Properties—A State-of-the-Art Review. Micro.

[114] Liang T., Ma Y., Jiang Z., Remón J., Zhou Y., Shi B. (2024). New insights into greener skin healthcare protection: Lignin nanoparticles as additives to develop natural-based sunscreens with high UV protection. Carbon Resour. Convers..

[115] Shao H., Zhang Y., Pan H., Jiang Y., Qi J., Xiao H., Zhang S., Lin T., Tu L., Xie J. (2022). Preparation of flexible and UV-blocking films from lignin-containing cellulose incorporated with tea polyphenol/citric acid. Int. J. Biol. Macromol..

[116] Ko Y., Kwon G., Choi H., Lee K., Jeon Y., Lee S., Kim J., You J. (2023). Cutting Edge Use of Conductive Patterns in Nanocellulose-Based Green Electronics. Adv. Funct. Mater..

[117] Haidari H., Kopecki Z., Sutton A.T., Garg S., Cowin A.J., Vasilev K. (2021). pH-Responsive “Smart” Hydrogel for Controlled Delivery of Silver Nanoparticles to Infected Wounds. Antibiotics.

[118] Yabuki A., Shiraiwa T., Fathona I.W. (2016). pH-controlled self-healing polymer coatings with cellulose nanofibers providing an effective release of corrosion inhibitor. Corros. Sci..

[119] Mi R., Li T., Dalgo D., Chen C., Kuang Y., He S., Zhao X., Xie W., Gan W., Zhu J. (2020). A Clear, Strong, and Thermally Insulated Transparent Wood for Energy Efficient Windows. Adv. Funct. Mater..

[120] Xia R., Zhang W., Yang Y., Zhao J., Liu Y., Guo H. (2021). Transparent wood with phase change heat storage as novel green energy storage composites for building energy conservation. J. Clean. Prod..

[121] Wang S., Li L., Zha L., Koskela S., Berglund L.A., Zhou Q. (2023). Wood xerogel for fabrication of high-performance transparent wood. Nat. Commun..

[122] Zhou J., Xu W. (2025). Phase-change thermal storage transparent wood based on optical reversibility. Ind. Crops Prod..

[123] Li Y., Fu Q., Rojas R., Yan M., Lawoko M., Berglund L. (2017). Lignin-Retaining Transparent Wood. ChemSusChem.

[124] Nagarajan V., Mohanty A.K., Misra M. (2016). Perspective on Polylactic Acid (PLA) based Sustainable Materials for Durable Applications: Focus on Toughness and Heat Resistance. ACS Sustain. Chem. Eng..

[125] Matuszek K., Kar M., Pringle J.M., Macfarlane D.R. (2023). Phase Change Materials for Renewable Energy Storage at Intermediate Temperatures. Chem. Rev..

[126] Okolie O., Kumar A., Edwards C., Lawton L.A., Oke A., McDonald S., Thakur V.K., Njuguna J. (2023). Bio-Based Sustainable Polymers and Materials: From Processing to Biodegradation. J. Compos. Sci..

[127] Chen H., Ma Y., Sheng X., Chen Y. (2024). Achieving heat storage coatings from ethylene vinyl acetate copolymers and phase change nano-capsules with excellent flame-retardant and thermal comfort performances. Prog. Org. Coatings.

[128] Liu H., Wang X., Wu D. (2019). Innovative design of microencapsulated phase change materials for thermal energy storage and versatile applications: A review. Sustain. Energy Fuels.

[129] Fang G., Tang F., Cao L. (2014). Preparation, thermal properties and applications of shape-stabilized thermal energy storage materials. Renew. Sustain. Energy Rev..

[130] Khadiran T., Hussein M.Z., Zainal Z., Rusli R. (2015). Encapsulation techniques for organic phase change materials as thermal energy storage medium: A review. Sol. Energy Mater. Sol. Cells.

[131] Huang Y., Stonehouse A., Abeykoon C. (2023). Encapsulation methods for phase change materials—A critical review. Int. J. Heat Mass Transf..

[132] Liu K., Yuan Z.F., Zhao H., Shi C.H., Zhao F. (2023). Properties and applications of shape-stabilized phase change energy storage materials based on porous material support—A review. Mater. Today Sustain..

[133] Wang J., Shen M., Liu Z., Wang W. (2022). MXene materials for advanced thermal management and thermal energy utilization. Nano Energy.

[134] Hemath M., Mavinkere Rangappa S., Kushvaha V., Dhakal H.N., Siengchin S. (2020). A comprehensive review on mechanical, electromagnetic radiation shielding, and thermal conductivity of fibers/inorganic fillers reinforced hybrid polymer composites. Polym. Compos..

[135] Safarkhani M., Far B.F., Huh Y., Rabiee N. (2023). Thermally Conductive MXene. ACS Biomater. Sci. Eng..

[136] Awan H.T.A., Abdah M.A.A.M., Mehar M., Walvekar R., Chaudhary V., Khalid M., Khosla A. (2024). MXene-polymer hybrid composites for advanced energy storage: Insights into supercapacitors and batteries. J. Energy Storage.

[137] Li D., Zhuang B., Chen Y., Li B., Landry V., Kaboorani A., Wu Z., Wang X.A. (2022). Incorporation technology of bio-based phase change materials for building envelope: A review. Energy Build..

[138] Reyez-Araiza J.L., Pineda-Piñón J., López-Romero J.M., Gasca-Tirado J.R., Arroyo Contreras M., Jáuregui Correa J.C., Apátiga-Castro L.M., Rivera-Muñoz E.M., Velazquez-Castillo R.R., Pérez Bueno J.d.J. (2021). Thermal Energy Storage by the Encapsulation of Phase Change Materials in Building Elements—A Review. Materials.

[139] Abdullatif Alshuhail L., Shaik F., Syam Sundar L. (2023). Thermal efficiency enhancement of mono and hybrid nanofluids in solar thermal applications—A review. Alexandria Eng. J..

[140] Ali H.M., Rehman T., Arıcı M., Said Z., Duraković B., Mohammed H.I., Kumar R., Rathod M.K., Buyukdagli O., Teggar M. (2024). Advances in thermal energy storage: Fundamentals and applications. Prog. Energy Combust. Sci..

[141] Patel D., Wei W., Singh H., Xu K., Beck C., Wildy M., Schossig J., Hu X., Hyun D.C., Chen W. (2023). Efficient and Secure Encapsulation of a Natural Phase Change Material in Nanofibers Using Coaxial Electrospinning for Sustainable Thermal Energy Storage. ACS Sustain. Chem. Eng..

[142] Ko H., Kang D.-G., Rim M., Koo J., Lim S.-I., Jang E., Yu D., Yoo M.-J., Kim N., Jeong K.-U. (2022). Heat managing organic materials: Phase change materials with high thermal conductivity and shape stability. Polym. Chem..

[143] Wijanarko N.P., Daniarta S., Kolasiński P. (2025). A Systematic Review of Biopolymer Phase Change Materials for Thermal Energy Storage: Challenges, Opportunities, and Future Direction. Energies.

[144] Sivanathan A., Dou Q., Wang Y., Li Y., Corker J., Zhou Y., Fan M. (2020). Phase change materials for building construction: An overview of nano-/micro-encapsulation. Nanotechnol. Rev..

[145] Liao S., Zhou X., Chen X., Li Z., Yamashita S., Zhang C., Kita H. (2024). Development of Macro-Encapsulated Phase-Change Material Using Composite of NaCl-Al_2_O_3_ with Characteristics of Self-Standing. Processes.

[146] Shah K.W. (2018). A review on enhancement of phase change materials—A nanomaterials perspective. Energy Build..

[147] Dmitruk A., Raźny N., Wu T., Serdechnova M., Naplocha K., Blawert C. (2024). PEO-coated aluminum alloys with good thermal conductivity for TES applications. Adv. Ceram. Coatings Energy Appl..

[148] Jiang J., Cao Y., Li G., Geng L., Zhang X., Zhao J., Liu C. (2025). Using Industrial Mining Solid Waste through Conversion to Phase-Change Materials for Thermal Energy Storage. Chempluschem.

[149] Ait Laasri I., Es-sakali N., Charai M., Mghazli M.O., Outzourhit A. (2024). Recent progress, limitations, and future directions of macro-encapsulated phase change materials for building applications. Renew. Sustain. Energy Rev..

[150] Shen Z., Qin M., Xiong F., Zou R., Zhang J. (2023). Nanocellulose-based composite phase change materials for thermal energy storage: Status and challenges. Energy Environ. Sci..

[151] Alam T.E., Dhau J.S., Goswami D.Y., Stefanakos E. (2015). Macroencapsulation and characterization of phase change materials for latent heat thermal energy storage systems. Appl. Energy.

[152] Ajdary R., Tardy B.L., Mattos B.D., Bai L., Rojas O.J. (2021). Plant Nanomaterials and Inspiration from Nature: Water Interactions and Hierarchically Structured Hydrogels. Adv. Mater..

[153] Jyothibasu J.P., Wang R.-H., Tien Y.-C., Kuo C.-C., Lee R.-H. (2022). Lignin-Derived Quinone Redox Moieties for Bio-Based Supercapacitors. Polymers.

[154] Atinafu D.G., Wi S., Yun B.Y., Kim S. (2021). Engineering biochar with multiwalled carbon nanotube for efficient phase change material encapsulation and thermal energy storage. Energy.

[155] Suchorowiec K., Paprota N., Pielichowska K. (2024). Aerogels for Phase-Change Materials in Functional and Multifunctional Composites: A Review. Materials.

[156] Tyagi V.V., Kaushik S.C., Tyagi S.K., Akiyama T. (2011). Development of phase change materials based microencapsulated technology for buildings: A review. Renew. Sustain. Energy Rev..

[157] Al Shannaq R., Farid M.M. (2015). Microencapsulation of phase change materials (PCMs) for thermal energy storage systems. Advances in Thermal Energy Storage Systems.

[158] Feizatidou A., Binas V., Kartsonakis I.A. (2025). Green Synthesis of Core/Shell Phase Change Materials: Applications in Industry and Energy Sectors. Energies.

[159] (2016). Standard Test Method for Enthalpies of Fusion and Crystallization by Differential Scanning Calorimetry.

[160] (2016). Plastics—Differential Scanning Calorimetry (DSC)—Part 1: General Principles.

[161] (2013). Standard Test Method for Thermal Conductivity of Solids by Means of the Guarded Compara-tive-Longitudinal Heat Flow Technique.

[162] (2009). Hygrothermal Performance of Buildings—Calculation and Presentation of Climatic Data—Part 3: Calculation of a Driving Rain Index for Vertical Surfaces.

[163] (2013). Standard Test Method for Thermal Diffusivity by the Flash Method.

[164] (2015). Plastics—Determination of Thermal Conductivity and Thermal Diffusivity—Part 2: Transient Plane Heat Source (TPS) Method.

[165] Liu P., Chen X., Li Y., Cheng P., Tang Z., Lv J., Aftab W., Wang G. (2022). Aerogels Meet Phase Change Materials: Fundamentals, Advances, and beyond. ACS Nano.

[166] Yang D., Tu S., Chen J., Zhang H., Chen W., Hu D., Lin J. (2023). Phase Change Composite Microcapsules with Low-Dimensional Thermally Conductive Nanofillers: Preparation, Performance, and Applications. Polymers.

[167] An X., Hu J., Wang S., Hou Y., Zou W. (2024). Characterization and optimization of desulfurized construction gypsum modified with functionalized nanocellulose. Alexandria Eng. J..

[168] Kalidasan B., Pandey A.K., Saidur R., Samykano M., Tyagi V.V. (2023). Nano additive enhanced salt hydrate phase change materials for thermal energy storage. Int. Mater. Rev..

[169] Jeong S.-G., Lee J.-H., Seo J., Kim S. (2014). Thermal performance evaluation of Bio-based shape stabilized PCM with boron nitride for energy saving. Int. J. Heat Mass Transf..

[170] Kumar K.R., Raghutu R.K., Venkata Padma D., Sastry S.V.A.R., Hemalatha R. (2025). Data-driven approaches to sustainable phase change material selection in latent heat storage systems. Int. J. Energy Water Resour..

[171] Coskun B., Temel U.N. (2025). Investigation of biochar performance for phase change material integration in building applications: Effects of raw material type and pyrolysis parameters. Eur. Phys. J. Plus.

[172] Hekimoğlu G., Sarı A., Arunachalam S., Arslanoğlu H., Gencel O. (2021). Porous biochar/heptadecane composite phase change material with leak-proof, high thermal energy storage capacity and enhanced thermal conductivity. Powder Technol..

[173] Yadav A., Samykano M., Pandey A.K., Natarajan S.K., Vasudevan G., Muthuvairavan G., Suraparaju S.K. (2024). Sustainable phase change material developments for thermally comfortable smart buildings: A critical review. Process Saf. Environ. Prot..

[174] Islam A., Pandey A.K., Sharma K., Bhutto Y.A., Saidur R., Buddhi D. (2024). Enhancing thermo-physical properties of hybrid nanoparticle-infused medium temperature organic phase change materials using graphene nanoplatelets and multiwall carbon nanotubes. Discov. Mater..

[175] Kumar R., Thakur A.K., Gupta L.R., Gehlot A., Sikarwar V.S. (2024). Advances in phase change materials and nanomaterials for applications in thermal energy storage. Environ. Sci. Pollut. Res..

[176] Abdolahimoghadam M., Rahimi M. (2024). New hybrid nano- and bio-based phase change material containing graphene-copper particles hosting beeswax-coconut oil for solar thermal energy storage: Predictive modeling and evaluation using machine learning. Energy.

[177] Brzęczek-Szafran A., Gwóźdź M., Brun N., Wysokowski M., Matuszek K. (2025). A Roadmap for Biomass-Driven Development of Sustainable Phase Change Materials. ChemSusChem.

[178] Rashid F.L., Al-Obaidi M.A. (2023). Recent innovations and developments concerning the beeswax as phase change material for thermal energy storage: A review. J. Therm. Anal. Calorim..

[179] Khalifelu S.S., Hamid N., Rahimi-Ahar Z., Seyedjabedar N., Oroujzadeh A., Babapoor A., Seyfaee A. (2024). Application of microencapsulated phase change materials for controlling exothermic reactions. Rev. Chem. Eng..

[180] Liang C., Wang X., Hao Y., Zhang Y., Jiang L., Tang B., Song Y. (2025). A comprehensive review of form-stable phase change materials in cold chain logistics: Encapsulation strategies, thermal conductivity enhancement, and applications. J. Mater. Chem. A.

[181] Cao H., Li Y., Xu W., Yang J., Liu Z., Bai L., Yang W., Yang M. (2022). Leakage-Proof Flexible Phase Change Gels with Salient Thermal Conductivity for Efficient Thermal Management. ACS Appl. Mater. Interfaces.

[182] Zhang H., Mai J., Li S., Althakafy J.T., Liu H., Alanazi A.K., El-Bahy S.M., Li X., Wu X., Wang R. (2022). Multi-functional phase change materials with anti-liquid leakage, shape memory, switchable optical transparency and thermal energy storage. Adv. Compos. Hybrid Mater..

[183] Liu Q., Zhang J., Liu J., Sun W., Xu H., Liu C. (2023). Self-healed inorganic phase change materials for thermal energy harvesting and management. Appl. Therm. Eng..

[184] Zhang J., Xu Y., Li X., Li H., Yao C., Chen S., Xu F. (2022). Leak-free, high latent heat and self-cleaning phase change materials supported by layered cellulose/Fe_3_O_4_ skeleton for light-to-thermal energy conversion. Energy Convers. Manag..

[185] Ahankari S., Paliwal P., Subhedar A., Kargarzadeh H. (2021). Recent Developments in Nanocellulose-Based Aerogels in Thermal Applications: A Review. ACS Nano.

[186] Pang Y., Sun J., Zhang W., Lai C., Liu Y., Guo H., Zhang D. (2024). Green, recyclable and high latent heat form-stable phase change composites supported by cellulose nanofibers for thermal energy management. Int. J. Biol. Macromol..

[187] Xing Y., Li W., Zhao Y., Yuan W., Ni C., Ye G., Yong Q., Zhang X., Sun Q., Bi H. (2025). Fabrication of multistage phase change nanocellulose composites with robust mechanical property and high thermal storage capacity. J. Energy Storage.

[188] Huang J., Su J., Weng M., Xiong L., Wang P., Liu Y., Lin X., Min Y. (2022). An innovative phase change composite with high thermal conductivity and sensitive light response rate for thermal energy storage. Sol. Energy Mater. Sol. Cells.

[189] Niu L., Li X., Zhang Y., Yang H., Feng J., Liu Z. (2022). Electrospun Lignin-Based Phase-Change Nanofiber Films for Solar Energy Storage. ACS Sustain. Chem. Eng..

[190] Heng Y., Feng N., Liang Y., Hu D. (2020). Lignin-retaining porous bamboo-based reversible thermochromic phase change energy storage composite material. Int. J. Energy Res..

[191] Lee J.J.C., Sugiarto S., Ong P.J., Soo X.Y.D., Ni X., Luo P., Hnin Y.Y.K., See J.S.Y., Wei F., Zheng R. (2022). Lignin-g-polycaprolactone as a form-stable phase change material for thermal energy storage application. J. Energy Storage.

[192] Liang Q., Pan D., Zhang X. (2023). Construction and application of biochar-based composite phase change materials. Chem. Eng. J..

[193] Lv L., Huang S., Zhou H. (2024). Performance investigation of biochar/paraffin composite phase change materials in latent heat storage systems: Feasibility of biochar as a thermal conductivity enhancer. J. Energy Storage.

[194] Yadav A., Samykano M., Pandey A.K., Kareri T., Kalidasan B. (2024). Optimizing thermal properties and heat transfer in 3D biochar-embedded organic phase change materials for thermal energy storage. Mater. Today Commun..

[195] Bordoloi U., Das D., Kashyap D., Patwa D., Bora P., Muigai H.H., Kalita P. (2022). Synthesis and comparative analysis of biochar based form-stable phase change materials for thermal management of buildings. J. Energy Storage.

[196] Guo Y., Guo X., Yin X., Zhang X., Hu S., Zhang Y., Yang H. (2025). Thermally driven memory flexible phase change hydrogel for solar energy efficient building thermal management. Sol. Energy Mater. Sol. Cells.

[197] Tan J., Luo S., Ji W., Li Y., Li L., Cheng X. (2022). Phase-changing hydrogels incorporated with copper sulfide-carbon nanotubes for smart thermal management and solar energy storage. J. Energy Storage.

[198] Song M., Wang L., Shao F., Xie H., Xu H., Yu W. (2023). Thermally induced flexible phase change hydrogels for solar thermal storage and human thermal management. Chem. Eng. J..

[199] Chen Y., Meng Y., Zhang J., Xie Y., Guo H., He M., Shi X., Mei Y., Sheng X., Xie D. (2024). Leakage Proof, Flame-Retardant, and Electromagnetic Shield Wood Morphology Genetic Composite Phase Change Materials for Solar Thermal Energy Harvesting. Nano-Micro Lett..

[200] Hanif F., Imran M., Zhang Y., Jia Z., Lu X., Lu R., Tang B. (2023). Form-Stable Phase Change Material with Wood-Based Materials as Support. Polymers.

[201] Li C., Tang H. (2024). Phase change material window for dynamic energy flow regulation: Review. Renew. Sustain. Energy Rev..

[202] Liu H., Wang X., Wu D. (2017). Fabrication of Graphene/TiO_2_/Paraffin Composite Phase Change Materials for Enhancement of Solar Energy Efficiency in Photocatalysis and Latent Heat Storage. ACS Sustain. Chem. Eng..

[203] Xie H., Zhao Y., Ma Y., Wen B., Zhao L., Han B., Li Z., Deng Q., Zhang K. (2024). Thermal conductive enhanced phase change composites with high latent-heat for constant temperature thermal management. J. Energy Storage.

[204] Xiao C., Wu X., Dong X., Ye G., Zhang G., Yang X. (2021). Ultrareliable Composite Phase Change Material for Battery Thermal Management Derived from a Rationally Designed Phase Changeable and Hydrophobic Polymer Skeleton. ACS Appl. Energy Mater..

[205] Fu T., Wang W., Fang G. (2024). Thermal properties and applications of form-stable phase change materials for thermal energy storage and thermal management: A review. Energy Storage.

[206] Zhang C., Zheng X., Xie N., Fang Y., Zhang Z., Gao X. (2023). Form-stable flexible composite multi-phase change material with high latent heat and enhanced thermal conductivity for thermal management. J. Energy Storage.

[207] Su Y., Shen J., Chen X., Xu X., Shi S., Wang X., Zhou F., Huang X. (2024). Bio-based eutectic composite phase change materials with enhanced thermal conductivity and excellent shape stabilization for battery thermal management. J. Energy Storage.

[208] Wang R., Feng Y., Li D., Li K., Yan Y. (2024). Towards the sustainable production of biomass-derived materials with smart functionality: A tutorial review. Green Chem..

[209] Chalivendula S.R., Tarigonda H. (2025). Recent Advances in Organic Phase Change Materials for Thermal Energy Storage: A Review on Sustainable Development Applications. Int. J. Thermophys..

[210] Srivastav D., Patil N.D., Shukla P.C. (2025). Bio-based phase change materials for battery thermal management: A numerical investigation for a single Li-ion cell. Appl. Therm. Eng..

[211] Guo J., Ding H., Xie J., Wang S., Huang Q., Liu C., Nie B. (2025). Bio-based, flexible, and electrically insulating phase change materials for advanced battery thermal management. J. Energy Storage.

[212] Wang T., Jiang Y., Huang J., Wang S. (2018). High thermal conductive paraffin/calcium carbonate phase change microcapsules based composites with different carbon network. Appl. Energy.

[213] Aiswarya V., Das S., James A., Saha S.K., Saha B.B. (2025). An experimental investigation on the solar thermal energy storage potential of form stabilized paraffin microcapsules. J. Environ. Chem. Eng..

[214] Um J.H., Ahn C.-Y., Kim J., Jeong M., Sung Y.-E., Cho Y.-H., Kim S.-S., Yoon W.-S. (2018). From grass to battery anode: Agricultural biomass hemp-derived carbon for lithium storage. RSC Adv..

[215] Wang X., Huang Y.-T., Liu C., Mu K., Li K.H., Wang S., Yang Y., Wang L., Su C.-H., Feng S.-P. (2019). Direct thermal charging cell for converting low-grade heat to electricity. Nat. Commun..

[216] Pulingam T., Appaturi J.N., Parumasivam T., Ahmad A., Sudesh K. (2022). Biomedical Applications of Polyhydroxyalkanoate in Tissue Engineering. Polymers.

[217] Giubilini A., Bondioli F., Messori M., Nyström G., Siqueira G. (2021). Advantages of additive manufacturing for biomedical applications of polyhydroxyalkanoates. Bioengineering.

[218] Zhang Y., Yu L., Zhang X.-D., Wang Y.-H., Yang C., Liu X., Wang W.-P., Zhang Y., Li X.-T., Li G. (2023). A smart risk-responding polymer membrane for safer batteries. Sci. Adv..

[219] He L., Wang M., Zhang X., Liu X., Luo Y., Chen Y., Fan Y. (2024). The confinement effect on phase change materials by physicochemical structure of wood-based materials. Ind. Crops Prod..

[220] Li Y., Wang B., Zhang W., Zhao J., Fang X., Sun J., Xia R., Guo H., Liu Y. (2022). Processing wood into a phase change material with high solar-thermal conversion efficiency by introducing stable polyethylene glycol-based energy storage polymer. Energy.

[221] Lam E., Hemraz U.D. (2021). Preparation and Surface Functionalization of Carboxylated Cellulose Nanocrystals. Nanomaterials.

[222] Paksoy H., Şahan N., Konuklu Y. (2022). Encapsulation of Phase Change Materials. Encyclopedia of Energy Storage.

[223] Zhou W., Wang W., Shi H., Zeng X., Li J., Pang Y., Chang Y.C., Sun R., Ren L. (2023). Multiway Softness Polyurethane Elastomeric Composite with Enhanced Thermal Conductivity and Application as Thermal Interface Materials. Adv. Mater. Technol..

[224] Wei Y., Jiang S., Li X., Li J., Dong Y., Shi S.Q., Li J., Fang Z. (2021). “green” Flexible Electronics: Biodegradable and Mechanically Strong Soy Protein-Based Nanocomposite Films for Human Motion Monitoring. ACS Appl. Mater. Interfaces.

[225] Song Y., Li B., Chen H., Yu Z. (2024). Research progress of absorbable stents. Int. J. Med. Sci..

[226] Lu W., Yu A., Dong H., He Z., Liang Y., Liu W., Sun Y., Song S. (2023). High-performance palmityl palmitate phase change microcapsules for thermal energy storage and thermal regulation. Energy.

[227] Szymanski P., Paluch R. (2024). Experimental investigation on heat pipes supported by soy wax and lauric acid for electronics cooling. J. Energy Storage.

[228] Yuan K., Chen Q., Qin M., Gao S., Wang Q., Gao S., Xiong F., Lv Y., Zou R. (2024). Micro/Nano Encapsulated Phase Change Materials: Preparation, Principle, and Emerging Advances in Medical Field. Adv. Funct. Mater..

[229] Wu H., Yang S., Li J., Ma T., Yang K., Liao T., Feng W., Zhou B., Yong X., Zhou K. (2024). Current status and challenges of shape memory scaffolds in biomedical applications. MedComm—Biomater. Appl..

